# The isolated HEPN domain of SACSIN exhibits RNA-binding activity

**DOI:** 10.1007/s00018-026-06369-w

**Published:** 2026-08-27

**Authors:** Lisa Longo, Irene Mariani, Antonella Cusimano, Rita Carrotta, Elsa Zacco, Roberta Vazzana, Giuseppe Cappelli, Maria R. Mangione, Maria A. Costa, Nicola Cuscino, Rosa Passantino, Silvia Vilasi, Alessia Gallo, Vincenzo Martorana, Roberto Giambruno

**Affiliations:** 1 Institute of Biophysics (IBF), National Research Council (CNR), Palermo, Italy; 2 Institute for Biomedical Research and Innovation (IRIB), National Research Council (CNR), Palermo, Italy; 3 Italian Institute of Technology – IIT, Genoa, Italy; 4 Department of Research, IRCCS-ISMETT (Istituto Mediterraneo Per I Trapianti E Terapie Ad Alta Specializzazione), Palermo, Italy

**Keywords:** ARSACS, SACSIN, RNA-binding proteins, HEPN, RNA–protein interactions

## Abstract

Autosomal Recessive Spastic Ataxia of Charlevoix–Saguenay (ARSACS) is a neurodegenerative disorder caused by mutations in the *SACS* gene, though the molecular function of its protein product, SACSIN, remains elusive. Therapeutic strategies for ARSACS are limited, mostly due to the exceptionally large size of SACSIN (~ 520 kDa), which precludes conventional gene therapy and standard molecular delivery methods. Over 200 pathogenic variants, including missense, nonsense and frameshift mutations, have been identified. Regardless of variant type, patients uniformly exhibit cerebellar ataxia, spasticity and peripheral neuropathy. Among them, patients with homozygous frameshift or missense mutations within the SACSIN higher eukaryotes and prokaryotes nucleotide-binding (HEPN) domain, suggesting that the alteration of the functional role of the SACSIN HEPN domain could be linked to the manifestation of ARSACS symptoms. Here, we show that the SACSIN HEPN domain can bind RNA in overexpression, corroborating the hypothesis that SACSIN functions as an RNA-binding protein. The interaction of the SACSIN HEPN domain with the RNA regulates the cellular compartmentalization of this domain as well as its propensity to form protein condensates. Conversely, an ARSACS mutated version of the SACSIN HEPN domain (F4574C) showed an impaired RNA-binding activity, a nuclear mis-localization and the predisposition to form protein condensates. Overall, our study unveils an unknown molecular function of the SACSIN HEPN domain that, if formally confirmed also for the full-length SACSIN, can pave the way for innovative therapeutic approaches for ARSACS patients.

**Graphical abstract:**

Created in BioRender. Vazzana, R. (2025) https://BioRender.com/rjn557o.

**Figure:**
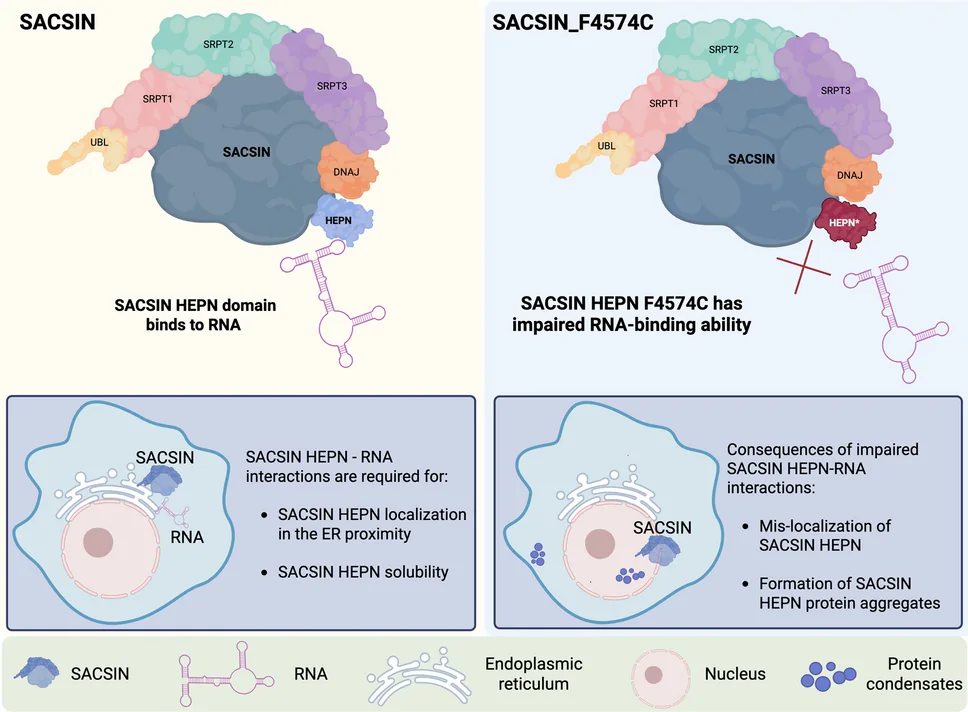

**Supplementary Information:**

The online version contains supplementary material available at https://doi.org/10.1007/s00018-026-06369-w.

## Introduction

Autosomal Recessive Spastic Ataxia of Charlevoix–Saguenay (ARSACS) is caused by mutations in the *SACS* gene [1, 2], which encodes for the multidomain protein SACSIN. Starting from the protein amino terminus, SACSIN possess one ubiquitin-like domain (UBL); three SACSIN internal repeats (SIRPT1, SIRPT1 and SIRPT3) containing three functional subrepeat regions (SR1, SR2 and SR3); one J-domain (DNAJ) and one higher eukaryotes and prokaryotes nucleotide-binding (HEPN) domain [3, 4]. The presence of these domains have been associated to the putative co-chaperone function of SACSIN in the cellular protein quality control [5]. Nevertheless, the molecular functions of SACSIN remains still elusive, due to its exceptionally large size that is incompatible with standard biochemical assays. Cells and mice depleted or with a reduced expression of SACSIN show disrupted proteostasis, aberrant alterations of the mitochondrial functions and cytoskeleton organization, particularly with the abnormal disorganization and bundling of intermediate filaments [6–10]. While the formation of highly ordered bundles of intermediate filaments can be considered a distinctive and early feature of ARSACS cells, the alteration of proteostasis and mitochondrial functions may be secondary consequences of the absence of SACSIN biological functions as they are common to many neurodegenerative disorders [11, 12].

So far, more than 200 mutations have been identified in ARSACS patients throughout the coding region of the *SACS* gene, including missense, nonsense and frameshift (both insertion and deletion) mutations [13]. Irrespective of the type of mutation, these patients show core clinical features such as cerebellar ataxia, spasticity and peripheral neuropathy, although there is considerable variability in the age of onset, severity and additional neurological features among individuals [4, 13]. Among them, ARSACS patients with homozygous frameshift or missense mutations in the SACSIN HEPN domain (N4463fs, Y4490S, E4511fs, N4549D, F4574C), which is exactly at the C-terminus of the protein [14–17]; thus, suggesting that the manifestation of ARSACS symptoms may also be connected to the absence or the altered functions of the HEPN domain.

The HEPN domain, present both in prokaryotes and eukaryotes, was initially reported to bind to nucleotides and serve as substrate-binding subunit of minimal nucleotide-transferases (MNT) [18]. Afterwards, all proteins containing a HEPN domain were grouped into the HEPN family, which is a class of RNA-binding proteins (RBPs). Most of them, having an endoribonuclease activity, belong to a subset named HEPN RNase [19]. HEPN RNases contain a conserved RΦxxxH motif within their HEPN domain that constitutes a metal-independent endoRNase active site [19, 20]. Other HEPN family proteins, such as the human SACSIN, lacking the RΦxxxH motif have been suggested to function as non-catalytic RBPs [20].

The concept that SACSIN may potentially bind to cellular RNA transcripts through its HEPN domain led us to hypothesize that alterations in SACSIN RNA-binding properties could be linked to several molecular defects observed in ARSACS patient cells.

Taking advantage of in silico*, *in vitro and in cells experiments, we are proving for the first time the ability of the SACSIN HEPN domain to bind to RNA transcripts. Specifically, we discovered that HEPN binds to both coding and non-coding RNA transcripts. SACSIN HEPN RNA-binding activity seems to have a specific role in the proximity of the endoplasmic reticulum (ER), where it interacts with the 7SL RNAs composing the signal recognition particle (SRP) complex (*e. g.* RN7SL1). The SRP is a ribonucleoprotein complex recognizing and targeting new proteins to the ER for their subsequent cellular trafficking [21].

Moreover, the interaction with the RNA is necessary to compartmentalize the SACSIN HEPN domain in close proximity to the ER and to regulate its propensity to form protein condensates. Protein condensates are reversible membraneless assemblies of proteins forming in cells through liquid–liquid phase separation process (LLPS) [22, 23]. The RNA can either act as a scaffold promoting LLPS or as negative regulator for the formation of protein condensates [24–26]. Here, we found out that the SACSIN HEPN domain forms protein condensates when its RNA substrates are not available.

## Results

### The HEPN domain of SACSIN binds RNA

The ability of SACSIN to bind RNA has not been experimentally tested, although the SACSIN HEPN domain can bind to guanosine triphosphate (GTP) with low micromolar affinity [27, 28].

**Fig. 1:**
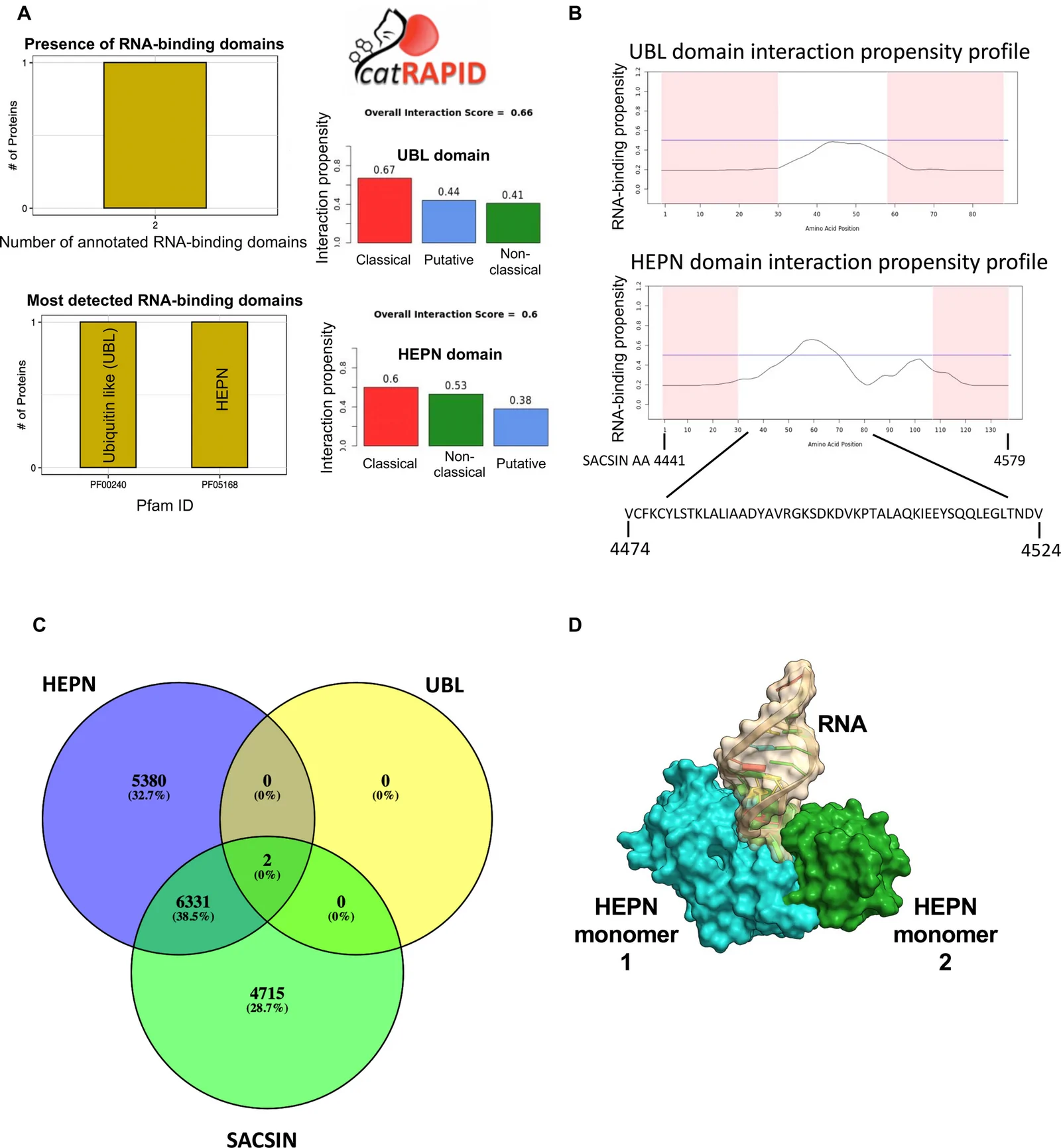
In silico predictions of SACSIN HEPN—RNA interactions. **A** Left. *cat*RAPID prediction of the interaction propensity of SACSIN toward cellular RNA transcripts. Two SACSIN domains (UBL and HEPN) are prone to bind to RNA. Right. Interaction propensity scores calculated by the *cat*RAPID algorithm for the UBL (upper part) and HEPN (lower part) domain. A positive interaction propensity is considered when Z-score > 0.5. **B** Interaction propensity profile of SACSIN UBL (upper part) and HEPN (lower part) domain calculated trough the *cat*RAPID algorithm. For the UBL domain, no specific binding regions were detected within the domain. The HEPN domain, instead, harboured a region of 50 amino acids (SACSIN amino acids (AA) 4474- 4524) with high interaction propensity with RNA transcripts. **C** Venn diagram of the human protein-coding RNAs predicted by *cat*RAPID as putative interactors of either SACSIN, the UBL or the HEPN domain. Only protein-coding RNAs with a Z-score > 1 were considered. **D** AlphaFold 3 prediction of the HEPN domain in its dimeric form interacting with a predicted 32 nucleotides GC-rich RNA sequence

We initially investigated the literature and found out that two independent studies, relying on the organic-aqueous phase separation methodology to unbiasedly purify and detect RBPs from cells, identified SACSIN among the proteins potentially able to interact with RNA transcripts in human cells [29, 30]. Consistently, we observed that SACSIN formed high molecular weight complexes upon UV-crosslinking (Supplementary Fig. 1A), as reported for other RBPs [31]. These findings support the possibility that SACSIN interacts with cellular RNAs. Therefore, we investigated SACSIN propensity to interact with cellular RNA transcripts through the well-established protein-RNA interaction prediction algorithm *cat*RAPID (Fig. 1) [32]. Interestingly, both the UBL and the HEPN domain, respectively located at the amino- and carboxy-terminus of SACSIN, were predicted to have the ability to bind to RNA, with an overall interaction propensity ≥ 0.6 (Fig. 1A). Neither domain showed a preference for a specific RNA-binding motif (Fig. 1A). For the UBL domain, no discrete amino acid region exceeded the RNA-binding threshold of 0.5. In contrast, the analysis of the HEPN domain identified a 50-amino acid region (V4474-V4524) as the main contributor to RNA binding, with the highest predicted interaction propensity > 0.65 (Fig. 1B).

We then used *cat*RAPID to predict the human RNA transcripts interacting with the full-length SACSIN, as well as its two putative RNA-binding domains, UBL and HEPN. Positive RNA–protein interactions were selected by applying a dynamic *cat*RAPID Z-score of > 1, as previously reported [32]. Full-length SACSIN and the HEPN domain showed the highest number of predicted protein-RNA interaction pairs, while only two RNAs were expected to associate with the UBL domain (Fig. 1C). Overall, suggesting that HEPN is the domain of SACSIN required for the interaction with RNAs. More than 38% of the putative interacting protein-coding RNAs were shared between full-length SACSIN and the HEPN domain (Fig. 1C), while the overlap among non-coding RNAs was lower (Supplementary Fig. 1B). We therefore focused on protein-coding RNAs with high interaction propensity (Z-score > 3.5) for both SACSIN and the HEPN domain and performed sequence alignment analysis using Clustal Omega Multiple Sequence Alignment algorithm [33]. Interestingly, we found the presence of a specific GC-rich consensus sequence containing putative quadruplex forming G-rich sequences [34], which was absent in the non-interacting RNAs (*cat*RAPID Z-score < 0) (Supplementary File 1). We analyzed the interaction of the predicted RNAs with the dimeric form of the HEPN domain, as we observed that the recombinant protein form stable dimers (Supplementary Fig. 1C), in line with the literature [27]. Additionally, we observed that the SACSIN HEPN domain specifically co-immunoprecipitate the endogenous SACSIN protein, supporting the hypothesis that also the endogenous protein can form protein dimers mediated by the HEPN domain (Supplementary Fig. 1D). We used AlphaFold 3 [35] to predict the structure of complexes between the HEPN dimer and a series of RNA sequences that were found by *cat*RAPID (Supplementary File 1). Notwithstanding the well-known AlphaFold 3 limitations in reliably predicting RNA structure, we were able to single out an interesting complex structure interaction with a GC-rich RNA sequences of 32 nucleotides containing two putative quadruplex forming G-rich sequences, indicating a specific interaction with the dimer (Fig. 1D and Supplementary Fig. 2A). We confirmed the stability and specificity of the HEPN-RNA interactions by molecular dynamics simulation, testing the stability of the complex along a 500 ns simulation. To investigate whether the HEPN domain recognizes specific RNA sequence features, we repeated the analysis using shorter 20-nucleotide RNA sequences, comparing a GC-rich RNA devoid of putative quadruplex forming G-rich sequences with a G-rich RNA predicted to form G-quadruplex structures (Supplementary Fig. 2A). Molecular dynamics simulations showed that the SACSIN HEPN domain interacted with the 20-nucleotide GC-rich RNA in a manner comparable to that observed for the 32-nucleotide GC-rich RNA. The G-rich RNA sequence of the same length showed, instead, a weak and non-specific interaction with the HEPN dimer, suggesting that G-quadruplex-forming sequences are not preferentially recognized by the SACSIN HEPN domain. In this latter case, the RNA molecule was displaced from the initial position and visited various regions of the dimer surface (Supplementary Fig. 2A). The interacting RNA localized within a hydrophilic cleft present at the monomer–monomer interface of the HEPN dimer (Fig. 1D and Supplementary Fig. 2A), mirroring the interaction binding mode of the SACSIN HEPN domain with GTP, previously observed by NMR spectroscopy [27].

**Fig. 2:**
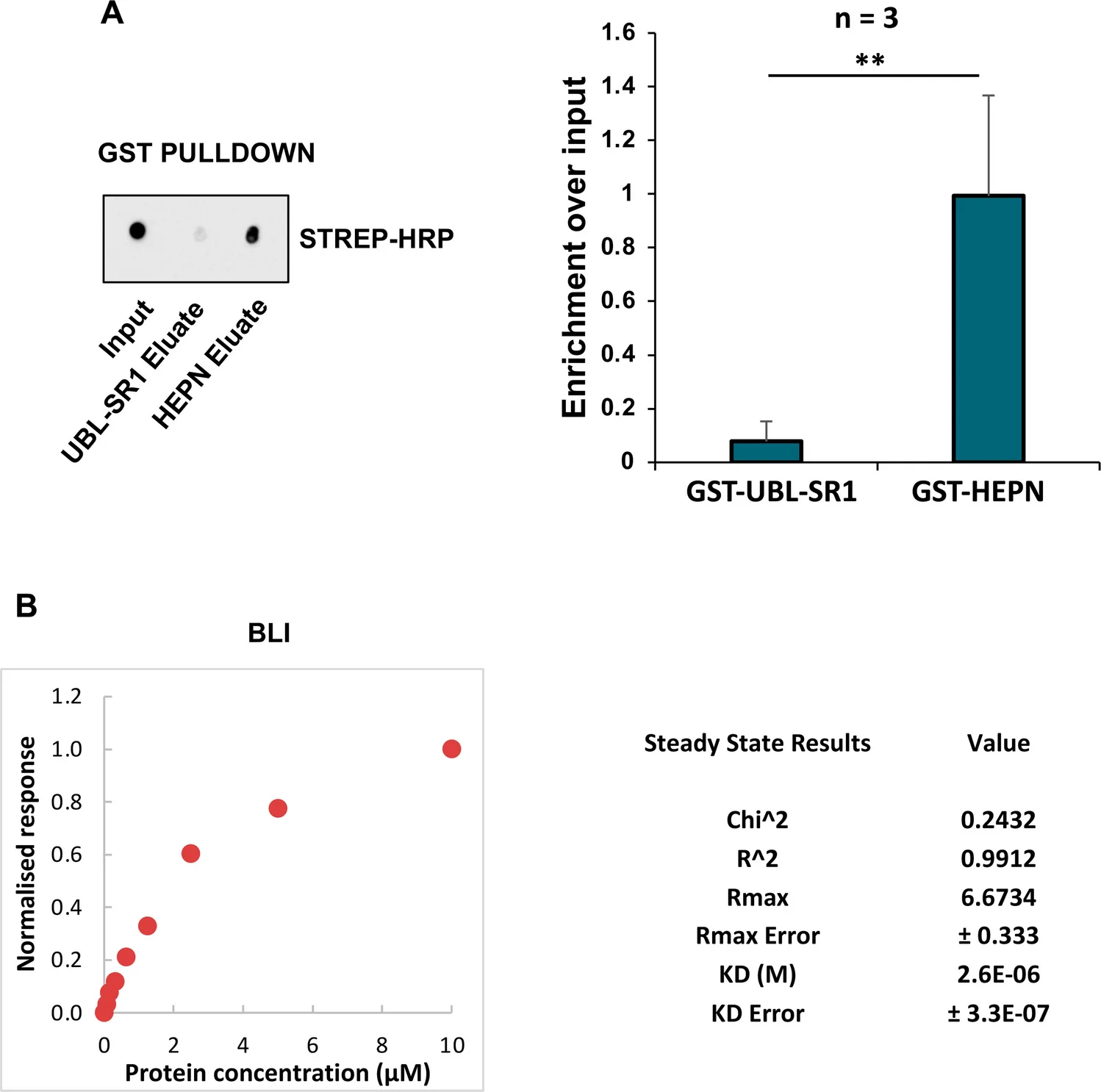
In vitro experiments to demonstrate SACSIN HEPN RNA-binding ability. **A** GST-pulldown of recombinant GST-tagged proteins with the synthetic biotinylated GC-rich RNA sequence. The data showed the average and the standard deviation of three biological replicates (*n* = 3) and the relative statistics (Two-sided T-Test *P* value ** = < 0.01. **B** BLI experiments to assess the K_D_ of GST-HEPN with the synthetic biotinylated GC-rich RNA sequence. Data were collected from three independent experiments and the average results are reported. The results were normalized by setting the highest value of the binding response to 1

We validated the interaction in vitro through GST-pulldown assays with recombinant GST-tagged SACSIN HEPN and UBL-SR1 proteins [27] and the synthetic biotinylated GC-rich RNA oligo of 32 nucleotides retrieved from *cat*RAPID predictions (Supplementary Fig. 2B and Supplementary File 1). The biotinylated RNA oligo was efficiently pulled down by GST-HEPN, while no interaction was detected with the GST-UBL-SR1 (Fig. 2A), confirming *cat*RAPID algorithm predictions. We then produced the recombinant HEPN domain devoid of GST-tag and measured its affinity for the RNA oligo using biolayer interferometry (BLI). The HEPN domain bound the predicted RNA target with a dissociation constant (K_D_) of 2.6 ± 0.3 µM (Fig. 2B), demonstrating that this domain recognizes the RNA with measurable affinity. Instead, under equal assay conditions, it was not possible to obtain any binding curve with a non-GC-rich RNA oligo of similar length, implying a non-detectable interaction between the HEPN domain and this RNA (Supplementary Fig. 3A). No significant changes in the secondary structure of either the protein or the GC-rich RNA oligo were observed by circular dichroism (CD) (Supplementary Fig. 3B and C). Likewise, no significant variations were observed in the protein tertiary structure monitored by the intrinsic fluorescence emission of the HEPN domain in the presence of RNA (Supplementary Fig. 3D). Overall, indicating that no structural perturbation occurs upon binding.

### The SACSIN HEPN domain localizes in the proximity of the ER, where it interacts with the SRP complex

**Fig. 3:**
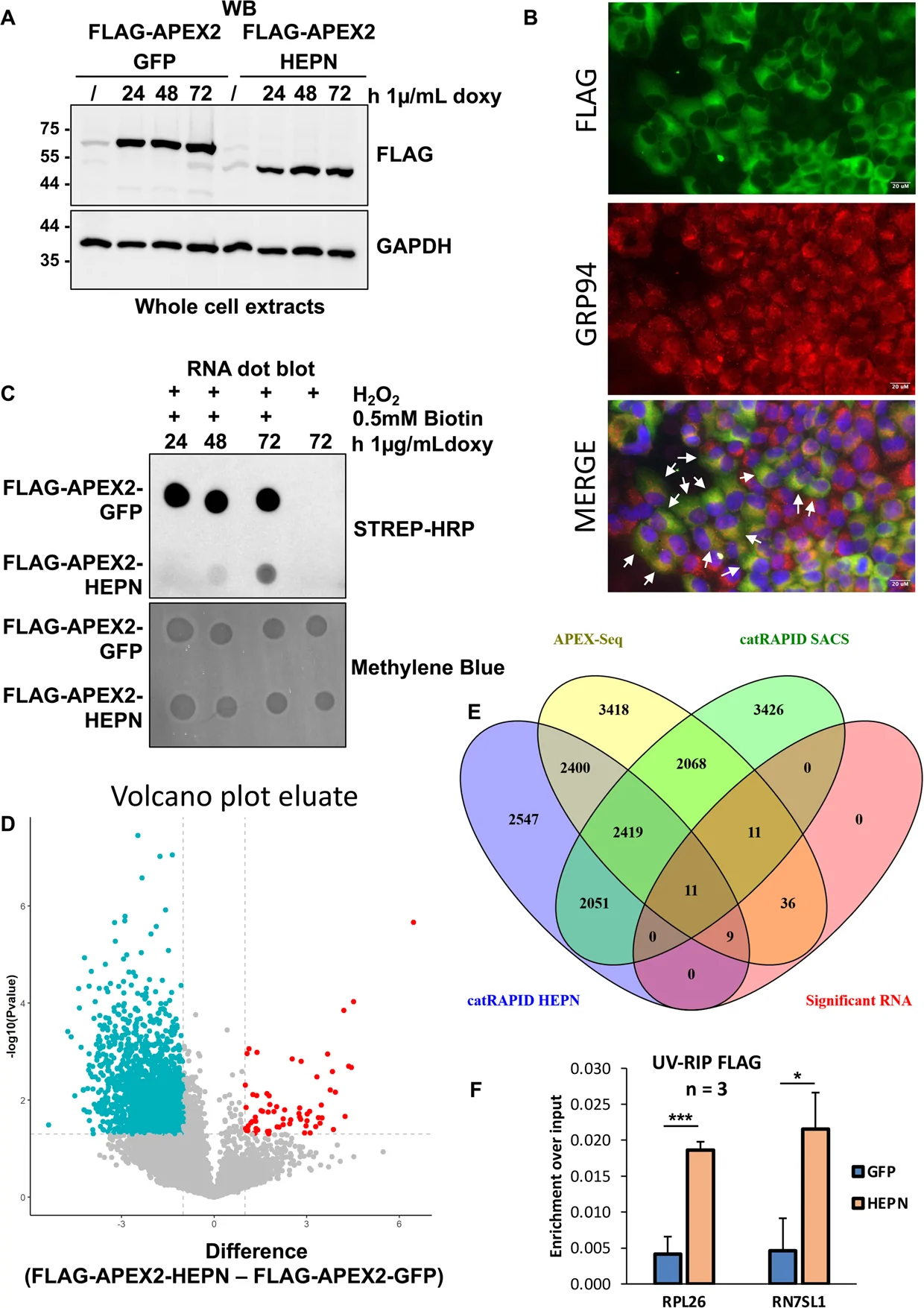
Confirmation of SACSIN HEPN RNA-binding activity and identification of RNA targets. **A** Expression of FLAG-APEX2-GFP and FLAG-APEX2-HEPN over time in response to doxycycline administration. GAPDH was used as loading control. The displayed Fig. is the representative image of one of the three performed biological replicates (*n* = 3). **B** Immunofluorescence analysis of Hela Flp-In™ T-Rex cells expressing FLAG-APEX2-HEPN, upon 72 h 1 µg/mL doxycycline administration. In green, the signal for FLAG-APEX2-HEPN while in red the protein GRP94, which is a chaperone protein located in the endoplasmic reticulum (ER). The white arrows indicated the cells where we observed a co-localization of the two proteins, confirming that FLAG-APEX2-HEPN accumulated in the ER-proximity. The displayed Fig. is the representative image of one of the three performed biological replicates (*n* = 3). **C** RNA dot blot analysis of the biotinylated RNAs recovered in response to the biotinylation reaction for the FLAG-APEX2-GFP and FLAG-APEX2-HEPN proteins at different time of doxycycline administration. Methylene blue staining was used as loading control of the RNAs spotted into the nitrocellulose membrane. The displayed Fig. is the representative image of one of the three performed biological replicates (*n* = 3). **D** Volcano plot analysis of streptavidin pulldown eluate derived from FLAG-APEX2-HEPN and FLAG-APEX2-GFP cells. RNA transcripts significantly enriched (log_2_ Fold change (Fc) > 1; two-sided T-Test *P* value < 0.05) in FLAG-APEX2-HEPN and FLAG-APEX2-GFP cells are colored in red and blue, respectively. **E** Venn diagram analysis of i) the significant RNA transcripts associated with FLAG-APEX2-HEPN compared to FLAG-APEX2-GFP identified by APEX-Seq (Significant RNA; red ellipse); ii) all the RNA transcripts found associated with FLAG-APEX2-HEPN by APEX-Seq (APEX-Seq; yellow ellipse); iii) RNA transcripts predicted to associate with SACSIN (*cat*RAPID SACSIN; green ellipse); iv) RNA transcripts predicted to associate with SACSIN HEPN domain (*cat*RAPID HEPN; violet ellipse). **F** UV-RNA Immunoprecipitation (UV-RIP)-FLAG of HeLa Flp-In.™ T-REx cells expressing either FLAG-APEX2-GFP or –HEPN, in response to 72 h of 1 µg/mL doxycycline administration. We confirmed that the RNA transcript coding for RPL26 and the non-coding RNA RN7SL1 were interacting with the immunopurified FLAG-APEX2-HEPN, in agreement with the APEX-Seq data. The data showed the average ± the standard deviation of three biological replicates (*n* = 3) and the relative statistics (Two-sided T-Test *P* value * = < 0.05; *** = < 0.001)

We sought to identify the cellular RNA transcripts bound by the HEPN domain in human cells using the APEX-Seq strategy [36, 37]. This proximity biotinylating system enables the identification of the RNA interactors of a specific protein in living cells, without the use of crosslinking agents [38]. We fused FLAG-APEX2 to the N-terminus of the HEPN domain as well as to a control eGFP, generating two fusion proteins of 46 and 60 kDa, respectively. The APEX2-fusion proteins were expressed from a tetracycline-inducible mammalian expression vector integrated in a single active genomic locus of HeLa Flp-In™ TRex cells; hence, permitting a homogenous and basal expression level of the exogenous FLAG-APEX2-tagged proteins. Upon doxycycline administration, the expression of FLAG-APEX2-GFP and FLAG-APEX2-HEPN was comparable and remained stable over time (Fig. 3A). In line with the expression of the endogenous SACSIN, FLAG-APEX2-HEPN localized to the cytoplasm. Interestingly, we observed that the protein was also present in the proximity of the endoplasmic reticulum (ER), especially after 72 h of doxycycline administration (Fig. 3B, Supplementary Fig. 4 and Supplementary Fig. 5). In contrast, FLAG-APEX2-GFP remained widely distributed within the cellular compartments (Supplementary Fig. 4). FLAG-APEX2-GFP was promiscuously associated with RNA transcripts, as previously reported [37]. FLAG-APEX2-HEPN, instead, began to interact with cellular RNA transcripts only after 48 h of doxycycline administration (Fig. 3C). No biotinylated RNA signal was detected after 24 h of doxycycline administration, as well as in the absence of biotin (Fig. 3C). To identify the RNA transcripts bound by FLAG-APEX2-HEPN, we performed APEX-Seq in biological triplicates in cells expressing either FLAG-APEX2-HEPN or FLAG-APEX2-GFP, after 72 h of doxycycline administration (Supplementary Fig. 6 A). The resulting biotinylated RNAs were affinity purified using streptavidin beads (Supplementary Fig. 6B) and sequenced through the Illumina next generation sequencing platform. The expression of FLAG-APEX2-HEPN did not alter the cellular transcriptome (Supplementary Fig. 7 A), with only 8 de-regulated RNA transcripts detected (Supplementary File 2). A total of 67 RNAs specifically and reproducibly interacted with FLAG-APEX2-HEPN compared to FLAG-APEX2-GFP (Fig. 3D and Supplementary File 2). These data were intersected with *cat*RAPID predictions to retrieve the *bona fide* list of HEPN RNA interactors. Among the interacting RNA transcripts, we found that 31 RNAs have a positive *cat*RAPID score (Z-score > 1.2) (Fig. 3E). The remaining 36 RNA transcripts were either absent from the *cat*RAPID library (26 RNA transcripts) or predicted not to interact with HEPN or SACSIN (Z-score < 1; 10 RNA transcripts). The 10 *cat*RAPID non-predicted RNA interactors were excluded because they (i) lack *cat*RAPID support and (ii) include transcriptionally upregulated genes (Ces1, S100p) (Supplementary File 2). For these reasons, we treated these RNAs as likely indirect or non-specific interactors for the purpose of defining a high-confidence set. In addition, we excluded the three mRNAs encoding mitochondrial proteins, as they might have derived from non-specific background labeling. The final list of *bona fide* HEPN interactors was composed of 54 RNA transcripts, comprising 27 mRNAs and 27 non-coding RNAs (Supplementary File 2). Gene ontology enrichment analysis conducted on either HEPN significant interactors or HEPN *bona fide* interactors revealed a significant enrichment of ER-localized RNAs and RNAs composing the SRP ribonucleoprotein complex, which regulates cellular protein trafficking [21] (Supplementary File 2). Finally, by ultraviolet-RNA immunoprecipitation (UV-RIP-FLAG), we confirmed that FLAG-APEX2-HEPN specifically interacted with the RNA transcripts coding for RPL26 and the non-coding RNA RN7SL1, which is the main core RNA component of the SRP complex [21] (Fig. 3F). In line, the endogenous SACSIN also significantly interacted with the RPL26 and RN7SL1 RNA transcripts (Supplementary Fig. 7B).

### The interaction with the RNA inhibits the formation of SACSIN HEPN condensates

**Fig. 4:**
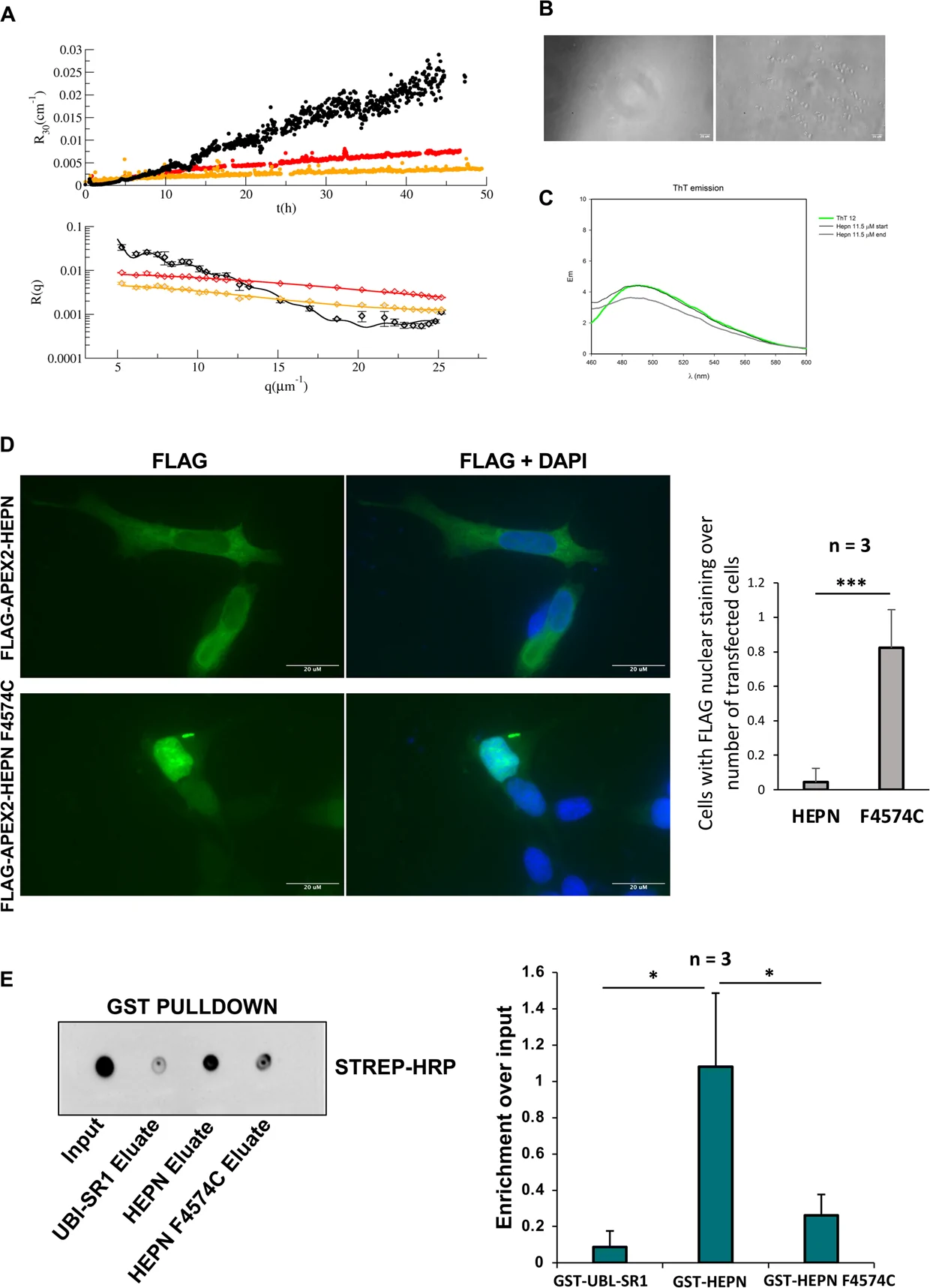
SACSIN HEPN RNA-binding properties regulates its propensity to form protein condensates. **A** Time-lapse Rayleigh ratio at θ = 30° of SACSIN HEPN domain alone (black curve) or mixed with 70 μg (low RNA, red curve) or 77 μg (high RNA, orange curve) of total RNA extracted from human cell lines, in all cases in the presence of 15% PEG6000 (top panel). R (q) MALS data for the three samples at the end of the kinetics, represented with the data best fitting functions (continuous lines) obtained according to the model reported in the material and methods section. HEPN forms larger condensates that did not occur in the presence of the RNA (bottom panel). **B** Representative bright-field images of SACSIN HEPN domain condensates using a wide-field microscope 40 × magnification. **C** Thioflavin (ThT) assay of SACSIN HEPN domain at the beginning and at the end of the kinetic. No significant variations in the ThT emission signal were observed between the initial and final samples, assessing that the HEPN condensates are not amyloid fibrils. **D** Immunofluorescence analysis of SACSIN knock-out SH-SY5Y cells transiently expressing FLAG-APEX2-HEPN or FLAG-APEX2-HEPN F4574C and fixed after 24 h from transfection with 4% PFA. Subsequently, immunofluorescence staining was performed with a-FLAG antibody. DAPI was used to stain the nuclei. The displayed Fig. is a representative image of the three performed biological experiments (*n* = 3). The quantification was performed in blind using 15 images taken for either FLAG-APEX2-HEPN or FLAG-APEX2-HEPN F4574C. The displayed data represent the average ± standard deviation of three biological replicates replicates (*n* = 3). The data are reported as ratio of transfected cells expressing FLAG nuclear staining over the total number of FLAG transfected cells (65 transfected cells for FLAG-APEX2-HEPN and 70 cells for FLAG-APEX2-HEPN F4574C). Statistic anaylysis was performed applying the Two-sided T-Test (Pvalue *** = < 0.001). **E** GST-pulldown of recombinant GST-tagged proteins with the synthetic biotinylated GC-rich RNA sequence. On the left, it is displayed a representative image of one of the three performed biological replicates (*n* = 3); on the right, the average ± standard deviation of the three performed experiments (*n* = 3) with the relative statistics (Two-sided T-Test *P* value * = < 0.05)

Since SACSIN HEPN domain binds to RNA, we sought to investigate whether it has a propensity to form RNA-dependent protein condensates [24–26]. Hence, we performed in vitro phase separation assays using the crowding agent polyethylene glycol PEG6000 [39, 40]. Light scattering at 30° revealed a time-dependent growth of HEPN condensates (Fig. 4A and B). Thioflavin-T staining confirmed that the final condensates lacked amyloid-like properties (Fig. 4C). Interestingly, static light scattering showed that the presence of total cellular RNA transcripts inhibited protein condensates formation in a dose-dependent manner (Fig. 4A). Furthermore, Multi Angle Light Scattering (MALS) analysis revealed RNA-dependent structural differences in the form factor of the protein condensates formed by HEPN alone compared to the ones formed by HEPN in the presence of RNA. The data recorded for HEPN alone were best defined by the presence of large spherical species with a diameter of ~ 2.6 μm, consistently with optical microscopy inspection of the sample where protein assemblies of microns size were detected (Fig. 4A and B). Whereas in the presence of RNA, much smaller spherical species with a maximum diameter of 360 nm were detected (Fig. 4A).

We next established a doxycycline-inducible system for live-cell imaging of GFP-HEPN and observed that the overexpressed protein was initially soluble and localized in the cytoplasm (Supplementary Fig. 7 C). Over time, it translocated to the nucleus, where it formed protein condensates (Supplementary Fig. 7D). In contrast, GFP-HEPN carrying the ARSACS-associated F4574C mutation was immediately mis-localized to the nucleus and formed protein condensates at early time points (Supplementary Fig. 7 C). Similar results were obtained in SACSIN knock-out SH-SY5Y and HeLa cell lines transiently overexpressing FLAG-APEX2-HEPN or FLAG-APEX2-HEPN F4574C (Fig. 4D, Supplementary Fig. 8 and 9A). Based on these results, we hypothesized that the F4574C mutation might impair the RNA-binding properties of SACSIN. Consistently, GST-HEPN F4574C showed reduced RNA-binding ability compared with the wild-type protein (Fig. 4E). However, we were unable to assess the RNA-binding properties of SACSIN HEPN F4574C in cells because the protein was sensibly less expressed than SACSIN HEPN (Supplementary Fig. 9B).

## Discussion

Our study provides the first direct evidence that the overexpressed SACSIN HEPN domain can bind cellular RNAs, revealing a previously uncharacterized molecular function that had been computationally predicted [20] but never experimentally investigated. Using the APEX2-tagged HEPN domain, we reported that the SACSIN HEPN domain interacts with RNAs localized in the ER proximity, including components of the SRP ribonucleoprotein complex [21], a critical mediator of ER-targeted protein translocation [41, 42]. The interaction with some ER-associated RNAs retrieved by APEX-Seq may partially reflect the ER-proximal localization of FLAG-APEX2-HEPN rather than the existance of direct HEPN-RNA interactions. However, the specific association of the RPL26 and RN7SL1 RNAs with both the SACSIN HEPN domain and the full-length SACSIN was orthogonally confirmed by UV-RIP assays. Notably, the interaction with the core component of the SRP complex, RN7SL1, raises the possibility that SACSIN may act as a local coordinator of nascent peptide recognition and ER trafficking. This hypothesis is compatible with the impaired trafficking of membrane and secreted proteins observed in ARSACS cells [8].

We further show that the SACSIN HEPN domain has the propensity to form protein condensates, a process that is inhibited by the interaction with the RNA. Circular dichroism and intrinsic fluorescence measurements indicate that the binding of the protein to the RNA did not necessarily alter the secondary structure or the global environment of the aromatic residues present in the SACSIN HEPN domain. Similar behavior has been reported for other RBPs, such as TDP-43, which binds specific RNA sequences without detectable changes in its secondary structure [43]. In this context, the inhibition of condensate formation observed in the presence of total cellular RNA is consistent with a mechanism in which the RNA modulates the phase separation of the SACSIN HEPN domain by interfering with multivalent intermolecular interactions.

Our data suggest a dual role for the RNA: i) a structural scaffold promoting proper subcellular localization near the ER; ii) a chaperone function preventing aberrant homodimeric interactions. Disruption of SACSIN HEPN RNA-binding ability, as exemplified by the F4574C mutation, leads to aberrant nuclear condensation, potentially progressing toward toxic protein aggregates. This mechanism echoes conceptual processes described for the pathogenic processes observed in other neurodegenerative disorders, including amyotrophic lateral sclerosis (ALS) and frontotemporal lobar degeneration (FTLD), where mutations in RNA-binding proteins such as FUS and TDP-43 drive pathological condensation and aggregation [44–46]. However, the SACSIN HEPN F4574C nuclear condensates were observed under overexpression conditions and their presence and composition in neurons expressing endogenous SACSIN mutants remain to be established.

Our data also raise important considerations regarding genotype–phenotype correlations in ARSACS patients. While most patients exhibit near-complete loss of SACSIN due to co-translational ubiquitination [47], individuals harbouring C-terminal mutations express truncated proteins [14] that may accumulate as non-functional protein condensates, potentially forming toxic aggregates. This observation offers an alternative perspective on how SACSIN biological functions may be altered in ARSACS patients with SACSIN C-terminal mutations, although thys hypothesis still needs to be validated in patient-derived cells.

Critically, although our findings establish that the SACSIN HEPN domain binds RNAs, several questions remain. The precise RNA targets and the structural determinants mediating SRP interaction require further characterization. Moreover, the long-term cellular consequences of SACSIN condensates, including their possible transition to irreversible aggregates, warrant in-depth investigation. The results obtained with the SACSIN HEPN domain will also need to be validated for the full-length SACSIN. For instance, the complete set of RNA transcripts bound by SACSIN in human cells remains to be determined, as well as understand whether SACSIN is able to bind RNAs alone or only as part of a ribonucleoprotein complex. Finally, it is important to highlight that the main conclusions of the proposed model should be verified in additional models such as ARSACS induced pluripotent stem cells (iPSC) derived-neurons [13, 48] and in vivo models [9].

In conclusion, our work not only uncovers a previously unappreciated molecular function for SACSIN but also suggest a potential link between an altered RNA-binding activity and disease-relevant cellular phenotypes present in ARSACS cells.

### Limitation of the study

Our findings are mainly addressing the molecular function of the SACSIN HEPN domain, while we could not collect the same experimental data with the full-length protein due to experimental limitations. We were able to demonstrate that SACSIN was specifically interacting with the RN7SL1 and RPL26 RNA transcripts. All the other biochemical and cellular data exclusively derived from overexpressed HEPN constructs rather than the endogenous full-length SACSIN. Working with the endogenous SACSIN is extremely challenging and we will require additional different experimental setup to address its RNA-binding properties. Moreover, some of SACSIN-RNA interactions may be tissue specific and part of our findings will have to be confirmed in neuronal differentiated cells as well as in ARSACS animal models.

## Materials and methods

### Reagents and plasmids

The following plasmids were purchased from Addgene: pDEST-cDNA5-FRT/TO-3*Flag-APEX2 N-term was a gift from Benjamin Blencowe (Addgene plasmid # 182925; http://n2t.net/addgene:182925; RRID: Addgene_182925); pGEX4T-HEPN was a gift from Kalle Gehring (Addgene plasmid # 75296; http://n2t.net/addgene:75296; RRID:Addgene_75296); pGEX6P1-Ubl-sr1(SIRPT1) was a gift from Kalle Gehring (Addgene plasmid # 75,298; http://n2t.net/addgene:75298; RRID:Addgene_75298). pRetroX GFP Mps1 wt was a gift from Floris Foijer (Addgene plasmid # 63702; http://n2t.net/addgene:63702; RRID: Addgene_63702).

The cDNA of the HEPN domain was PCR-amplified using the following primers:

Forward 5’-GGGGACAAGTTTGTACAAAAAAGCAGGCTCCGTTGGCAATCCAGTGGAAGC-3’; Reverse 5’- GGGGACCACTTTGTACAAGAAAGCTGGGTTTACACTTTTTGTTGCATAAAATTTTCAAGT- 3’.

The amplified cDNA was cloned into the pDonor201 through BP reaction of the gateway cloning strategy (11789020, ThermoFisher Scientific). The cDNA was then transferred by LR reaction of the gateway cloning strategy (11791020, ThermoFisher Scientific) into the pDEST-cDNA5-FRT/TO-3*Flag-APEX2 N-term.

For the generation of pRetroX GFP-HEPN, both pRetroX GFP Mps1 wt and pGEX4T-HEPN were digest with BamHI (BA3136S, New England Biolabs) and NotI (BA3189S, New England Biolabs). The digested products were run on an agarose gel and the respective linear bands of insert (HEPN) and backbone (pRetroX GFP) were extracted from gel using the Wizard(R) SV Gel and PCR Clean-up System (A9281, Promega). The bands were then ligated overnight using the T4 DNA ligase (M0202S, New England Biolabs).

The HEPN F4574C mutant was generated by Q5® Site-Directed Mutagenesis Kit (BE0552S, New England Biolabs) using the following primers:

Forward 5’-ACTTGAAAATtgtATGCAACAAAAAGTG– 3’; Reverse 5’ -TTTATTATGATACAGGCAGTAC– 3’.

All the plasmids have been sequence verified by the sanger sequencing service offered by Eurofins.

Synthetic RNA oligos of 32 nucleotides were synthesized by Eurofins according to the following sequences: Predicted HEPN interacting RNA: CGGGGGCGGCGGCGGCGGCGGCGGCAGCGGGG.

For the GST pulldown and BLI assays, the predicted HEPN interacting RNA was synthesized including a 5’ biotin.

### Cell lines

HeLa, HEK293T and HeLa Flp-In™ TREx cells were grown in DMEM with glucose and L-glutamine (ECM0103L, Euroclone), 100 U/mL penicillin and streptomycin (ECB3001D, Euroclone) and supplemented with either 10% fetal bovine serum (FBS) (ECS5000L, Euroclone) or tetracycline free FBS (ECS01822, Euroclone), respectively.

SACSIN knock-out SH-SY5Y cells, kindly provided by Prof. F. M. Santorelli, were grown as above described except that cultivation medium was complemented with 15% FBS and supplemented with non-essential amino acids (ECB3054D, Euroclone).

### APEX-Seq

Cells were incubated at 37 °C for 30 min with complete media containing 0.5 mM biotinyl-tyramide (SML2135, Merck Life Science). Afterwards, the medium was discarded and 1 mM of H_2_O_2_ (H1009, Merck Life Science) in PBS was provided for 1 min at room temperature followed immediately by 3 washes with freshly prepared quenching solution (10 mM sodium ascorbate (27688.235, VWR Chemicals), 5 mM Trolox (238813, Merck Life Science), 10 mM sodium azide (S2002, Merck Life Science), PBS 1x). Cells were then pelleted and RNA extracted using the Miny RNAse extraction kit (R1054, Zymo Research), following vendor’s instruction and including the in-column DNAse I digestion step.

The eluted material was analyzed by RNA-dot blot assay using a STREP-HRP conjugate to confirm the presence of biotinylated RNAs. For each sample, 60 μg of total RNA was diluted with 1 mL of Blocking Buffer (5 × Denhardt’s Solution (D9905-5ML, Merck Life Science) in PBS1x supplemented with RNAse inhibitor) and 10% of the solution was taken as input material. The remaining 90% was affinity purified rocking it for 1 h at room temperature in combination with 30 uL of MyOne C1 Streptavidin Dynabeads (65001, ThermoFisher Scientific), that were previously washed 3 times with Buffer 1 (0.5% sodium deoxycholate in PBS 1 × supplemented with RNAse inhibitor (BIO-65028, Meridian Bioscience) and equilibrated with Blocking Buffer for 30 min at room temperature on a nutator. Samples were then placed on a magnetic rack to separate the unbound fraction from the magnetic beads. Beads were subsequentially washed with four different washing buffers (Buffer 2: 6 M urea pH 8.0, 0.1% SDS in PBS; Buffer 3: 2% (w/v) SDS in PBS; Buffer 4: 750 mM NaCl, 0.5% (w/v) sodium deoxycholate, 0.1% (w/v) SDS, in PBS; Buffer 5: 150 mM NaCl, 0.5% (w/v) sodium deoxycholate, 0.1% (w/v) SDS, in PBS) to maintain only the biotinylated RNA bound to the beads. A total of 2 washes of 1 mL of each buffer was performed. At the end of the washes, the bound biotinylated RNA was extracted from the beads using the Quick-RNA MicroPrep Kit (R1050, Zymo Research) and the RNA was eluted with 14 μL of nuclease free water.

### RNA dot blot assay

RNA was spotted on hybound nitrocellulose membrane (GERPN203B, Merck Life Science) and let it adsorb into the membrane. Then, the RNA was UV-crosslinked to the membrane using the UV-stratalinker with one cycle of 2500 Kjoule. To reveal the presence of biotinylated RNA, the membrane was then stained with the Chemiluminescent Nucleic Acid Module kit (89880, ThermoFisher Scientific), following vendor’s instruction,

Afterwards, the membrane is stained for 5 min with 0.1% (w/v) Methylene Blue solution in 0.3 M sodium acetate (pH 5.2. The pH is adjusted with acetic acid) and de-stained with milliQ water.

### Library preparation and next-generation sequencing

RNA integrity was evaluated with the Agilent 4200 TapeStation System (Agilent Technologies Ltd). RNA-Seq libraries for Input samples were prepared starting from 500 ng of RNA using the Illumina TruSeq Stranded Total RNA (20020596, Illumina) and TruSeq RNA UD Indexes 24 Indexes-96 Samples (20020177, Illumina). Briefly, total RNA input was deprived of ribosomal RNA and fragmented before the synthesis of single and double strand cDNA, as previously described [49]. Then, double strand cDNA 3’ends were adenylated and Illumina indexing adapters were ligated. RNA-Seq libraries for HEPN-Elute and GFP-Elute samples were prepared using the NEBNext Ultra II Directional RNA Library Prep Kit for Illumina (BE7760S, New England Biolabs) and NEBNext Multiplex Oligos for Illumina (Index Primers Set 1, E7335, New England Biolabs). Briefly, eluted RNA was deprived of ribosomal RNA using the NEBNext rRNA depletion kit (BE7405L, New England Biolabs), fragmented before the synthesis of single and double strand cDNA and then adapters were ligated.

For both types of libraries, library size distribution and quality was evaluated on the Agilent 4200 TapeStation System (Agilent Technologies Ltd). Finally, libraries were pooled in equimolar concentration based on Qubit (Invitrogen) quantification, and sequencing was performed on a NextSeq™ 550 (Illumina) with 2 × 76 cycles, following the manufacturer’s instructions. Quality control of the raw sequencing data was done using FastQC (v0.11.9, Babraham Institute, Babraham, UK). Subsequently, Trimmomatic (v0.39) was employed to eliminate low-quality reads and conduct adapter trimming. The extracted RNA sequences were aligned with the human reference genome (hg19) using STAR (v2.7.0) (https://github.com/alexdobin/STAR/releases). Transcript abundances were quantified with RSEM (v1.3.3) (https://deweylab.github.io/RSEM/) and gene expression levels normalized to transcripts per million (TPM).

### UV-RIP

UV-RIP was performed as previously described [50, 51]. HeLa Flp-In™ TREx or SH-SY5Y cells were harvested, washed once with PBS 1 × and UV-crosslinked on ice with 2 cycles of irradiations at 100000 μJ/cm2. Cells were lysed with Lysis Buffer (0.5% NP-40; 0.5% NaDeoxycholate; cocktail protease inhibitors (11836153001, Roche); 25 U/mL RNAse inhibitor (BIO-65028, Meridian Bioscience); PBS 1x). The lysates were rocked on a wheel for 30 min at 4 °C and then treated with 30 U of Turbo DNAseI (AM2239, ThermoFisher Scientific) for 30 min at 37 °C in a Thermomixer shacking at 1100 rpm. DNA-digested lysates were subsequently centrifuged for 5 min at 1350 g to remove cellular debris. An aliquot of the supernatant (10%) was kept as input. The remaining collected supernatant (90%) was split in two fractions in fresh tubes and IgG (Millipore) and anti-SACSIN (Abcam) rabbit antibodies were added to each fraction, respectively. The final volume of the IP was brought to 500 μL with additional Lysis Buffer and the samples were rocked overnight at 4 °C. The day after, Sera-Mag SpeedBeads Protein A/G (17152104010150, Cytiva) were first washed 3 times in Lysis Buffer and then equilibrated 1:1 in the same buffer: 50 uL of the bead slurry were added to each tube. Samples were rocked for additional 3 h at 4 °C, to allow the antibody capture by the beads. Afterwards, beads were pelleted on a magnet, the supernatants discarded and the beads washed 4 times with Washing Buffer I (PBS supplemented with 1% NP-40, 0.5% NaDeoxycholate, 300 mM NaCl, cocktail protease inhibitor and 25 U/mL RNAse inhibitor). The beads were then resuspended in 100 μL RNAse-free water and treated again with the Turbo DNAseI for 30 min at 37 °C, in a Thermomixer at 1100 rpm. The 10% input material was also treated a second time with DNAseI. The dynabeads were then washed 4 times with Washing Buffer II (PBS supplemented with 1% NP-40, 0.5% NaDeoxycholate, 300 mM NaCl, 10 mM EDTA, cocktail protease inhibitor and 25 U/mL RNAse inhibitor). Finally, RNA-Lysis Buffer (Zymo Research) was added to the beads and the RNA extracted using the RNA-extraction kit (Zymo Research). Eventually, to remove residual genomic DNA, the RNA fractions were boiled for 2 min at 95 °C, followed by 5 min incubation in ice, and then put at 37 °C for 1 h in the presence of Turbo DNAseI. The same procedure was performed for the extraction of the RNA from the input material that is used to estimate the levels of the immuno-enrichment. The RNA was again extracted with the RNA extraction kit and then retro-transcribed to cDNA for RT-qPCR analysis. The values obtained for each immunopurifications were normalized over their respective input material and plotted in a histogram, as relative fold enrichment over the not immunopurified RNA.

### RT-qPCR analysis

The RNA was retro-transcribed into cDNA using the ImProm-IITM (A3800, Promega), according to the vendor’s instruction. The cDNA was then diluted 1:5 with water and 5% of the diluted material was analyzed by RT-qPCR analysis using the POWER SYBR GREEN PCR master mix (4367659, ThermoFisher Scientific). The values obtained for each immunoprecipitated RNAs were normalized over the respective input material and plotted in a histogram, as relative fold enrichment.

The following primers have been used for RT-qPCR analysis:

RPL26 Forward primer 5’ -GGCTAATGGCACAACTGTCCAC- 3’; Reverse primer 5’ -GGCGAGATTTGGCTTTCCGTTC- 3’.

RN7SL1 Forward primer 5’- CATCAATATGGTGACCTCCCG- 3’; Reverse primer 5’- GTTTTGACCTGCTCCGTTTC- 3’.

### Co-immunoprecipitation analysis

Whole cell extracts from HeLa Flp-In™ TREx cells were obtained using IP-buffer (50 mM Tris–HCl pH 7.5; 150 mM NaCl; 5 mM EDTA; 5 mM EGTA; 1% NP40; H_2_O) complemented with cocktail protease inhibitors (11836153001, Roche). The whole cell extracts (1 mg) were brought to a final volume of 500 µL with IP-buffer and 10% of the material was taken as input. The remaining 90% was immunopurified with 30 μL anti-FLAG M2 beads (M8823, Millipore) rocking the samples over night at 4 °C. The following day, beads were washed with 1 mL of IP-buffer and the bound material eluted with 50 μL 4 × Laemmli buffer + 100 mM b-mercaptoethanol, boiled 5 min at 95 °C and then collect the eluted material in a fresh tube. The input and eluted material were subjected to SDS-PAGE followed by WB analysis.

### Immunofluorescence analysis

Cells were fixed in 4% paraformaldehyde (12606, Cell Signaling Technology) dissolved in PBS for 10 min at room temperature, followed by 3 washes with PBS 1 × of 5 min each. Cells were then permeabilized by adding 0.1% TRITON-PBS for 10 min at R.T. and washed 3 times with PBS 1 × for 5 min each washing step. Aspecific sites were blocked with 2% BSA-PBS 1 × for 30 min at R.T., prior adding the anti-FLAG M2 (F1804, Merck Life Science) primary antibody diluted in 2% BSA-PBS 1 × for 2 h at R.T. After the incubation, 3 washes with PBS 1 × of 5 min each were performed, followed by the staining with the secondary antibody (Alexa 488 (A11029 and A11034, Invitrogen) or Alexa 555 (A31572, Invitrogen) for 1 h at R.T.. At the end, 3 × washes in PBS of 5 min each were performed and the coverslips mounted with antifade mounting medium with DAPI (EN53003M010, Enzo Life Sciences). For the co-immunostaining, the above-described protocol was performed simultaneously incubating the anti-FLAG rabbit (F7425, Merck) with either anti-Calreticulin mouse (sc-373863, Santa Cruz) or anti-GRP94 mouse (sc-1794, Santa Cruz). The respective secondary antibodies with two different fluorophores (Alexa 488 and Alexa 555) were also simultaneously provided. Images were acquired using the widefield Evident-Olympus IX83 microscope.

### Co-localization analysis

Co-localization analysis was performed using the public available *in-house* app [Vincenzo Martorana (2026). Pycoloc (v.1.0.0beta). GitHub. https://github.com/asmart64/pycoloc], that we wrote to overcome a numerical instability problem that we encountered using the coloc2 plugin of Fiji imageJ. The analysis is based on the evaluation of Manders coefficients and on the Costes randomization test.

A region of interest (ROI) is drawn around a chosen cell in the image for the quantification of the co-localization signal of the cell in the image and the test calculates the thresholded Manders’ coefficient tM1 and tM2, the Costes observed r (number of blocks = 100, PSF = 3 pixels), the Costes significance and the Monte Carlo pValue, as previously described [52, 53].

### Live-cell imaging

A TET-ON system for the tetracycline-inducible expression of HEK293T cells was generated by transfecting with ViaFECT (E4981, Promega) a mammalian expression plasmid expressing the transactivator rtTA3. Cells stably integrating the plasmid were selected through puromycin selection. These cells were then transiently transfected overnight with the pRetroX GFP-HEPN. The following day, the cultivation media was replaced with fresh medium supplemented with 1 μg/mL doxycycline to activate the TET-ON system. The expression of GFP-HEPN was acquired in live cells at the indicated time points, using the Evident-Olympus IX83 microscope.

### Protein extraction and Western Blot analysis

Proteins were extracted using RIPA Buffer (10 mM Tris–HCl pH 8.0, 1% NP-40, 150 mM NaCl, 0.1% SDS, 0.1% NaDeoxycholate, 1 mM EDTA) complemented with cocktail protease inhibitor (11836153001, Merck Life Science). Cells were lysed on ice for 15 min at 4 °C and then spun down at 15000 g for 15 min. The supernatant containing the protein extracts were transfer into a clean 1.5 mL tube and protein concentration quantified by Bio-Rad Protein Assay Dye (5000006, BIO-RAD Laboratories). For Western Blot analysis, 20 μg of protein whole cell extracts were resolved by SDS-PAGE gel and then transferred into a nitrocellulose membrane. The membrane was blocked with 5% milk dissolved in 0.05% TWEEN-TBS 1 × and then incubated over night at 4 °C with one of the following primary antibodies: anti-FLAG M2 (F1804, Merck Life Science); Anti-SACSIN antibody (EPR11864, Abcam); anti-GAPDH (14C10, Cell Signaling); anti-Vinculin (A2752, ABclonal). The following day, after three washing steps of 5 min with 0.05% TWEEN-TBS 1x, the membrane was incubated for 1 h with the HRP-conjugated secondary antibody (1706515 and 1706516, BIO-RAD Laboratories), washed for additional three washing steps of 5 min with 0.05% TWEEN-TBS 1 × and then revealed by chemiluminescence using the clarity western ECL substrate (1705061, BIO-RAD Laboratories) and recorded with the ChemiDocTM Imaging System (12003153, BIO-RAD Laboratories).

### GST-pulldown assay

To preserve the proper RNA-folding [54], the synthetic biotinylated RNA was boiled for 2 min at 95 °C and then put on ice for 5 min. Subsequently, the RNA was resuspended in the pulldown reaction buffer (PD-buffer: 2 mM MgCl_2_, 100 U/mL RNAse inhibitor, protease inhibitor, 0.05% BSA, 0.2% NP-40, PBS 1x) and let it stand for 20 min at room temperature. For the assay, 10 pmol of RNA was denatured and refolded in a final volume of 550 μL of PD buffer, according to the abovementioned protocol. Afterwards, 10% of the material was taken as input and the remaining 90% divided in two 1.5 mL tubes containing 20 μg of either GST-HEPN or GST-UBI-SR1, previously quantified by Coomassie staining into a final volume of 500 μL. Samples were rocked on a wheel for 1 h at room temperature and afterwards beads were pulled down by centrifugation and washed 3 times with PD-buffer and the bound RNA extracted with the Miny RNAse extraction kit (R1054, Zymo Research). The RNA was eluted in 30 μL of nuclease free water and 2 μL used for the RNA dot blot assay.

### *cat*RAPID analysis

*cat*RAPID prediction to determine the interaction propensity of SACSIN toward cellular RNA transcripts were conducted using the *cat*RAPID omics v2.1 webfold [32]. The interaction propensities of the amino acidic sequence of canonical full-length SACSIN (Q9NZJ4-1), the UBL (SACSIN amino acids 1–84) and HEPN (SACSIN amino acids 4441- 4579) domains were investigated against human protein-coding RNAs. A positive interaction propensity is considered when the Z-score was > 1.

### Generation of venn diagrams

Venn diagrams were generated using the following webtool: Oliveros, J.C. (2007–2015) Venny. An interactive tool for comparing lists with Venn's diagrams. https://bioinfogp.cnb.csic.es/tools/venny/index.html

### Molecular modeling

The structure of HEPN dimer in complex with the following RNA sequences were predicted using AlphaFold 3 [35]. We used 3 different RNA sequences:

- GC-rich RNA sequences of 32 nucleotides containing two putative quadruplex forming G-rich sequences: 5’ -CGGGGGCGGCGGCGGCGGCGGCGGCAGCGGGG – 3’;
- GC-rich RNA sequences of 20 nucleotides lacking putative quadruplex forming G-rich sequences: 5’ – GGGCCUGGAGCCGGAUCUAA- 3’.
- GC-rich RNA sequences of 20 nucleotides containing one putative quadruplex forming G-rich sequences: 5’—GGGGUGGGGGAGGACGCCGA—3’.

The presence of putative quadruplex forming G-rich sequences was assessed using the QGRS Mapper software [34] (https://bioinformatics.ramapo.edu/QGRS/index.php).

For the 20-nucleotide RNAs, that have a feasible simulated system size, we adopted the standard options for the Alphafold 3 webserver predictions, getting 5 different models for each input. We always chose the first in the ranking for running subsequent simulations.

For the analysis of the HEPN dimer in complex with the 32-nucleotide RNA, used for the in vitro assays, we explored a more thorough prediction effort: we installed the Alphafold 3 software in our server and ran repeated instances of the prediction processes to collect a grand total of 100 structures of the protein-RNA complex. Then, we used a python script to select the best candidate in terms of RNA–protein interaction and prediction quality, as previously reported [55], and selected the first ranking prediction to run a 500 ns MD simulation. We used the following empirical scoring weights for the above ranking: i) contact = 0.35 (RNA–protein contact multiplicity); ii) bsa = 0.25 (buried RNA–protein surface area); iii) dist = 0.15 (RNA–protein interface distance favorability); iv) quality = 0.25 (Alphafold 3 predicted interface quality).

The predictions were of good quality for the protein (ipTM = 0.94 and pTM = 0.95), but scarce for what concerns the RNA structures. However, the predicted structures of the 20- and 32- nucleotide RNAs were sufficiently good to run stable molecular dynamics simulations using Gromacs vers. 2021.3 [56]. The three complexes were solvated with TIP3 water molecules and 150 mM of KCl ions were added using CharmmGUI [57]. We used the Charmm36m force field [58] and the Particle Mesh Ewald to describe the electrostatic interactions. The structures were minimized for 1000 steps, then a 2.5 ns NVT simulation was performed while harmonically restraining the complex to the initial coordinates. Eventually, NPT simulations were performed using a timestep of 2 fs for 500 ns.

### Biolayer interferometry

Biolayer interferometry (BLI) experiments were performed using an Octet Red instrument (ForteBio, Inc., Menlo Park, CA) at 25 °C. Binding assays were carried out in 20 mM potassium phosphate buffer (pH 7.4) containing 150 mM KCl and 0.05% Tween-20. Streptavidin-coated biosensors were loaded with 0.5 µg/ml of biotinylated RNA aptamer and subsequently exposed to increasing concentrations of protein, ranging from 75 nM to 10 µM. Dissociation constants (K_D_) were determined by fitting the steady-state response (wavelength shift upon binding) as a function of protein concentration. Each assay was repeated three times, with measurements performed in triplicate. The BLI experiments were performed using the HEPN dimer in complex with either the GC-rich RNA of 32 nucleotides (sequence: 5’ -CGGGGGCGGCGGCGGCGGCGGCGGCAGCGGGG – 3’) or the non-GC-rich RNA of similar length (sequence: 5’ -AACCAACUUUCGAUCUCUUGUAGAUCUGUUCU −3’). The same protein dilutions were used for both RNAs but for the non-GC-rich RNA it was not possible to generate any binding curve, since no response was observed, implying a lack of interaction between HEPN and this RNA.

### Protein purification

*Escherichia coli* BL21-Gold (DE3) harbouring pGEX4T-HEPN plasmid, coding for the fusion protein GST-HEPN, or PGEX6P1-Ubl-sr1(SIRPT1), coding for GST-UBL-SR1, was grown at 37 °C in LB medium supplemented with 100 µg/mL ampicillin. When OD_600_ reached 0.6, the expression was induced by addition of 0.1 mM isopropyl 1-thio-β-d-galactopyranoside (IPTG) for 4 h at 30 °C. Upon harvesting, bacterial pellet was washed with PBS (10 mM Na_2_HPO_4_, 1.7 mM KH_2_PO_4_, 136 mM NaCl, 2.6 mM KCl) and resuspended with lysis buffer (PBS supplemented with 1 mg/mL lysozyme, cOmplete™ EDTA-free protease inhibitor mixture (11836170001, Roche), 5 mM MgCl_2_, 10 µg/ml DNase, 2 mM 1,4-dithiothreitol), and disrupted by sonication (Bandelin HD 2070) on ice. The supernatant was collected after centrifugation at 20.000 g, 4 °C for 30 min, filtered using 0.2 μm filter and rocked for 1 h at room temperature with Glutathione Sepharose 4B, previously equilibrated in PBS. After washing three times with PBS, the resin was stored at 4 °C and used for the GST-pulldown experiments. For the biophysical assays, the GST-tag of HEPN protein was removed by digestion with 1 U/mL thrombin protease (27084601, Cytiva) upon rocking overnight at room temperature. The mixture containing cleaved HEPN protein and thrombin was collected from the resin using PBS as elution buffer. The thrombin protease was removed using HiTrap™ Benzamidine FF column (17514302, Cytiva). Then, HEPN protein was eluted in two steps using PBS first and PBS-1 M (10 mM Na_2_HPO_4_, 1.7 mM KH_2_PO_4_, 1 M NaCl, 2.6 mM KCl) later. A further purification was obtained by gel filtration using a Superdex 75 10/300 gl column (17517401, Cytiva) on a FPLC ӒKTA Pure 25 (Cytiva), eluting with 20 mM phosphate buffer (10 mM Na_2_HPO_4_, 10 mM NaH_2_PO_4_, pH 7.4), 0.15 M NaCl, 5% (w/v) glycerol. The purity of the protein was verified by SDS-PAGE followed by Coomassie staining. Protein was stored at − 80 °C.

### Sample preparation for fluorescence and circular dichroism experiments

A 20 µM RNA solution was denatured at 95 °C in nuclease-free water for 2 min and then cooled on ice for 5 min. After cooling, it was diluted to obtain a 6 µM RNA sample in 20 mM phosphate buffer, 150 mM NaCl, 5% glycerol, RNase inhibitor (40 U/µL), and 2 mM MgCl₂.

After 20 min, the refolded RNA was mixed with the HEPN samples at the required concentrations to achieve the desired composition of both components, maintaining RNA-to-protein volume ratio of 1:2.

### Fluorescence spectroscopy

Fluorescence experiments were performed by using a JASCO FP6500 spectrofluorometer (JASCO Corporation, Tokyo, Japan) at T = 20 °C. Intrinsic fluorescence experiments were performed registering emission spectra after excitation at 295 nm (excitation slit width = 3 nm, emission slit width = 3 nm, response 2 s, scanning rate 100 nm/min) using a quartz cell with a path length of 1 cm.

Thioflavin-T (ThT) fluorescence (12 µM ThT, 20 °C; λ_exc = 450 nm, λ_em = 485 nm) was measured for ThT alone and after incubation with HEPN samples obtained at the start and at the end of the condensate-formation kinetics.

### Circular dichroism

CD spectroscopic measurements were made by using a JASCO J-810 spectrometer equipped with a temperature control unit. A quartz cell with a path length of 0.5 mm was used for recording at 20 °C the spectra (200–320 nm) of a sample containing 2 µM RNA in the absence or in the presence of 3 µM, 5 µM, 9 µM or 20 µM HEPN. Spectra were acquired with a 20 nm/min scan rate, band width 2 nm, response 16 s and data pitch 0.5 nm. CD spectra of the protein alone were also recorded in the same conditions for each concentration used. Samples were corrected using the respective blank.

### Multi angle light scattering (MALS)

Protein solutions were prepared at 23 μM in 10 mM phosphate buffer, 150 mM NaCl, and 5% (v/v) glycerol. Where indicated, RNA was added to the protein solution at 0.40 μg/μL (low RNA) or 0.44 μg/μL (high RNA). For measurements, 170 μL of the protein solution (with or without RNA) was mixed 1:1 with 170 μL of PEG6000 (30% w/w; Farmalabor) prepared in the same buffer, yielding a final volume of 340 μL. The final concentration upon mixing were: HEPN domain 11.5 μL, PEG6000 15% w/w, and RNA 0/0.20/0.22 μg/μL for the no-RNA, low-RNA, and high-RNA conditions, respectively. All solutions were handled under dust-free conditions and loaded into cylindrical quartz cells. Multi-angle light scattering measurements were performed on a Brookhaven Instruments BI-200SM goniometer equipped with a 20 mW He–Ne laser (λ = 632.8 nm) and a Brookhaven BI-9000 correlator. Cells were mounted in a thermostated compartment maintained at 20.0 °C (± 0.1 °C) via a recirculating bath.

Kinetic acquisition: Time-lapse scattering at ϑ = 30° was collected every 3 min for 2 days to monitor condensate formation kinetics.

Angular acquisition: For absolute form-factor analysis, the scattering intensity I(q) was measured at 27 angles, corresponding to q spanning in the range of 5.3 ÷ 25.2 µm⁻^1^. The magnitude of the scattering vector was defined as q = 4πn/λ sin(θ/2), with n the solvent refractive index. Taking *n* = 1.33 with λ = 632.8 nm and θ = 30°, the scattering vector is q≈6.8 μm^^−1^.

Measured intensities were converted to the absolute Rayleigh ratio R(q) using toluene as standard, with R_tol_ (λ = 632.8 nm) = 14 × 10^–6^ cm^−1^. The Rayleigh ratio of the sample at angle θ was obtained as R(q) = (I(q(θ)) sinθ/(I_tol_ x R_tol_, where I(q(θ)) and I_tol_ are the sample and the toluene scattering intensity respectively measured under identical optical setup. For dilute solutions, the angular dependence of the absolute intensity was analyzed using R(q) = kcMP(q), where k is the optical constant, c the solute concentration, M the intensity-weighted average molar mass of the scatterers, and P(q) the form factor. Spherical form factors were used to extract the gyration radius R_g_ and infer particle size. For HEPN alone in 15% PEG6000 data were best described by the contribution of two spherical species, one with R_g_ (1) = 440 nm and a second with R_g_ (2) = 2.6 μm, while the two samples with RNA were analysed by using a single spherical specie with R_g_ (low RNA) = 330 nm and R_g_ (high RNA) = 250 nm, respectively. The kinetics trace at θ = 30° (absolute intensity versus time) is reported in Fig. 4A (upper panel). Best-fit form factors for the three samples are shown as continuous lines in Fig. 4A, together with the corresponding absolute-scale MALS data (lower panel).

## Supplementary Information

Below is the link to the electronic supplementary material.

Supplementary figures (PDF 6169 KB)

Supplementary file1 (DOCX 320 KB)

Supplementary file2 (XLSX 20007 KB)

## Data Availability

RNA-Seq data were deposited in the GEO public repository database with the following number: GSE312176.

## References

[1] Bouchard JP, Barbeau A, Bouchard R, Bouchard RW (1978) Autosomal recessive spastic ataxia of charlevoix-saguenay. Can J Neurol Sci J Can Sci Neurol 5:61–69 647499

[2] Baets J et al (2010) Mutations in *SACS* cause atypical and late-onset forms of ARSACS. Neurology 75:1181–1188 10.1212/WNL.0b013e3181f4d86c 20876471

[3] Romano A et al (2013) Comparative analysis and functional mapping of *SACS* mutations reveal novel insights into sacsin repeated architecture. Hum Mutat 34:525–537 10.1002/humu.22269 PMC3629688 23280630

[4] Bagaria J, Bagyinszky E, An SSA (2022) Genetics of autosomal recessive spastic ataxia of charlevoix-saguenay (ARSACS) and role of sacsin in neurodegeneration. Int J Mol Sci 23:552 10.3390/ijms23010552 PMC8745260 35008978

[5] Parfitt DA et al (2009) The ataxia protein sacsin is a functional co-chaperone that protects against polyglutamine-expanded ataxin-1. Hum Mol Genet 18:1556–1565 10.1093/hmg/ddp067 PMC2667285 19208651

[6] Francis V et al (2022) The ARSACS disease protein sacsin controls lysosomal positioning and reformation by regulating microtubule dynamics. J Biol Chem 298:102320 10.1016/j.jbc.2022.102320 PMC9437860 35933016

[7] Duncan EJ et al (2017) Altered organization of the intermediate filament cytoskeleton and relocalization of proteostasis modulators in cells lacking the ataxia protein sacsin. Hum Mol Genet. 10.1093/hmg/ddx197 PMC5886247 28535259

[8] Romano LEL et al (2022) Multi-omic profiling reveals the ataxia protein sacsin is required for integrin trafficking and synaptic organization. Cell Rep 41:111580 10.1016/j.celrep.2022.111580 PMC9647044 36323248

[9] Aly KA, Moutaoufik MT, Zilocchi M, Phanse S, Babu M (2022) Insights into SACS pathological attributes in autosomal recessive spastic ataxia of Charlevoix-Saguenay (ARSACS). Curr Opin Chem Biol 71:102211 10.1016/j.cbpa.2022.102211 36126381

[10] Gentil BJ et al (2019) Sacsin, mutated in the ataxia ARSACS, regulates intermediate filament assembly and dynamics. FASEB J 33:2982–2994 10.1096/fj.201801556R 30332300

[11] Gan L, Cookson MR, Petrucelli L, La Spada AR (2018) Converging pathways in neurodegeneration, from genetics to mechanisms. Nat Neurosci 21:1300–1309 10.1038/s41593-018-0237-7 PMC6278826 30258237

[12] Wilson DM et al (2023) Hallmarks of neurodegenerative diseases. Cell 186:693–714 10.1016/j.cell.2022.12.032 36803602

[13] Salem IH et al (2025) ARSACS: clinical features, pathophysiology and iPS-derived models. Cerebellum (London, England) 24:24 10.1007/s12311-024-01777-9 39753868

[14] Tunca C et al (2024) Sacsin levels in PBMCs: a diagnostic assay for SACS variants in peripheral blood cells – a PROSPAX study. Mov Disord 39:2291–2297 10.1002/mds.30012 39314081

[15] Petrov I (2021) Novel mutation in <span class="SmallCaps" style="font-weight: normal;">SACS</span> gene in a patient with autosomal recessive spastic ataxia charlevoix-saguenay. Mov Disord Clin Pract 8:963–965 10.1002/mdc3.13216 PMC8354083 34405106

[16] Richter AM, Ozgul RK, Poisson VC, Topaloglu H (2004) Private SACS mutations in autosomal recessive spastic ataxia of Charlevoix-Saguenay (ARSACS) families from Turkey. Neurogenetics 5:165–170 10.1007/s10048-004-0179-y 15156359

[17] Raval MA et al (2024) Journey through autosomal-recessive spastic ataxia of Charlevoix–Saguenay: insights from a case series of seven patients–a single-center study and review of an Indian cohort. J Mov Disord 17:430–435 10.14802/jmd.24154 PMC11540545 39198013

[18] Grynberg M, Erlandsen H, Godzik A (2003) HEPN: a common domain in bacterial drug resistance and human neurodegenerative proteins. Trends Biochem Sci 28:224–226 10.1016/S0968-0004(03)00060-4 12765831

[19] Pillon MC, Gordon J, Frazier MN, Stanley RE (2021) HEPN RNases – an emerging class of functionally distinct RNA processing and degradation enzymes. Crit Rev Biochem Mol Biol 56:88–108 10.1080/10409238.2020.1856769 PMC7856873 33349060

[20] Anantharaman V, Makarova KS, Burroughs AM, Koonin EV, Aravind L (2013) Comprehensive analysis of the HEPN superfamily: identification of novel roles in intra-genomic conflicts, defense, pathogenesis and RNA processing. Biol Direct 8:15 10.1186/1745-6150-8-15 PMC3710099 23768067

[21] Costa EA, Subramanian K, Nunnari J, Weissman JS (2018) Defining the physiological role of SRP in protein-targeting efficiency and specificity. Science 359:689–692 10.1126/science.aar3607 PMC5970945 29348368

[22] Alberti S, Gladfelter A, Mittag T (2019) Considerations and challenges in studying liquid-liquid phase separation and biomolecular condensates. Cell 176:419–434 10.1016/j.cell.2018.12.035 PMC6445271 30682370

[23] Alberti S, Hyman AA (2021) Biomolecular condensates at the nexus of cellular stress, protein aggregation disease and ageing. Nat Rev Mol Cell Biol 22:196–213 10.1038/s41580-020-00326-6 33510441

[24] Maharana S et al (2018) RNA buffers the phase separation behavior of prion-like RNA binding proteins. Science 360:918–921 10.1126/science.aar7366 PMC6091854 29650702

[25] Wadsworth GM et al (2024) RNA-driven phase transitions in biomolecular condensates. Mol Cell 84:3692–3705 10.1016/j.molcel.2024.09.005 PMC11604179 39366355

[26] Roden C, Gladfelter AS (2021) RNA contributions to the form and function of biomolecular condensates. Nat Rev Mol Cell Biol 22:183–195 10.1038/s41580-020-0264-6 PMC7785677 32632317

[27] Kozlov G et al (2011) Structural basis of defects in the Sacsin HEPN domain responsible for autosomal recessive spastic ataxia of Charlevoix-Saguenay (ARSACS). J Biol Chem 286:20407–20412 10.1074/jbc.M111.232884 PMC3121481 21507954

[28] Li X et al (2015) High-throughput screening for ligands of the HEPN domain of Sacsin. PLoS ONE 10:e0137298 10.1371/journal.pone.0137298 PMC4569058 26366743

[29] Queiroz RML et al (2019) Comprehensive identification of RNA–protein interactions in any organism using orthogonal organic phase separation (OOPS). Nat Biotechnol 37:169–178 10.1038/s41587-018-0001-2 PMC6591131 30607034

[30] Urdaneta EC et al (2019) Purification of cross-linked RNA-protein complexes by phenol-toluol extraction. Nat Commun 10:990 10.1038/s41467-019-08942-3 PMC6397201 30824702

[31] Urdaneta EC, Beckmann BM (2020) Fast and unbiased purification of RNA-protein complexes after UV cross-linking. Methods 178:72–82 10.1016/j.ymeth.2019.09.013 31586594

[32] Armaos A, Colantoni A, Proietti G, Rupert J, Tartaglia GG (2021) Cat RAPID omics v2.0: going deeper and wider in the prediction of protein–RNA interactions. Nucleic Acids Res 49:W72–W79 10.1093/nar/gkab393 PMC8262727 34086933

[33] Madeira F et al (2024) The EMBL-EBI job dispatcher sequence analysis tools framework in 2024. Nucleic Acids Res 52:W521–W525 10.1093/nar/gkae241 PMC11223882 38597606

[34] Kikin O, D’Antonio L, Bagga PS (2006) QGRS Mapper: a web-based server for predicting G-quadruplexes in nucleotide sequences. Nucleic Acids Res 34:W676–W682 10.1093/nar/gkl253 PMC1538864 16845096

[35] Abramson J et al (2024) Accurate structure prediction of biomolecular interactions with AlphaFold 3. Nature 630:493–500 10.1038/s41586-024-07487-w PMC11168924 38718835

[36] Fazal FM et al (2019) Atlas of subcellular RNA localization revealed by APEX-Seq. Cell 178:473-490.e26 10.1016/j.cell.2019.05.027 PMC6786773 31230715

[37] Padrón A, Iwasaki S, Ingolia NT (2019) Proximity RNA labeling by APEX-Seq reveals the organization of translation initiation complexes and repressive RNA granules. Mol Cell 75:875-887.e5 10.1016/j.molcel.2019.07.030 PMC6834362 31442426

[38] Giambruno R, Nicassio F (2022) Proximity-dependent biotinylation technologies for mapping RNA-protein interactions in live cells. Front Mol Biosci 9:1062448 10.3389/fmolb.2022.1062448 PMC9702341 36452457

[39] Alfano C et al (2024) Molecular crowding: the history and development of a scientific paradigm. Chem Rev 124:3186–3219 10.1021/acs.chemrev.3c00615 PMC10979406 38466779

[40] André AAM, Spruijt E (2020) Liquid-liquid phase separation in crowded environments. Int J Mol Sci 21:5908 10.3390/ijms21165908 PMC7460619 32824618

[41] Pool MR, Stumm J, Fulga TA, Sinning I, Dobberstein B (2002) Distinct modes of signal recognition particle interaction with the ribosome. Science 297:1345–1348 10.1126/science.1072366 12193787

[42] Halic M et al (2006) Following the signal sequence from ribosomal tunnel exit to signal recognition particle. Nature 444:507–511 10.1038/nature05326 17086193

[43] Bhardwaj A, Myers MP, Buratti E, Baralle FE (2013) Characterizing TDP-43 interaction with its RNA targets. Nucleic Acids Res 41:5062–5074 10.1093/nar/gkt189 PMC3643599 23519609

[44] Lagier-Tourenne C, Polymenidou M, Cleveland DW (2010) TDP-43 and FUS/TLS: emerging roles in RNA processing and neurodegeneration. Hum Mol Genet 19:R46–R64 10.1093/hmg/ddq137 PMC3167692 20400460

[45] Zbinden A, Pérez-Berlanga M, De Rossi P, Polymenidou M (2020) Phase separation and neurodegenerative diseases: a disturbance in the force. Dev Cell 55:45–68 10.1016/j.devcel.2020.09.014 33049211

[46] Molliex A et al (2015) Phase separation by low complexity domains promotes stress granule assembly and drives pathological fibrillization. Cell 163:123–133 10.1016/j.cell.2015.09.015 PMC5149108 26406374

[47] Longo F et al (2021) Assessment of sacsin turnover in patients with ARSACS: implications for molecular diagnosis and pathogenesis. Neurology. 10.1212/WNL.0000000000012962 PMC8665432 34649874

[48] Martineau L et al (2026) Generation of eight human induced pluripotent stem cells lines from patients with Autosomal Recessive Spastic Ataxia of Charlevoix-Saguenay (ARSACS). Stem Cell Res 91:103894 10.1016/j.scr.2025.103894 41529449

[49] Gallo A et al (2025) Transcriptomic profiles of monocyte-derived macrophages exposed to SARS-CoV-2 VOCs reveal immune-evasion escape driven by delta. J Transl Med 23:151 10.1186/s12967-025-06158-2 PMC11796281 39905461

[50] Spadotto V et al (2020) PRMT1-mediated methylation of the microprocessor-associated proteins regulates microRNA biogenesis. Nucleic Acids Res 48:96–115 10.1093/nar/gkz1051 PMC6943135 31777917

[51] Blatter M et al (2023) RNA binding induces an allosteric switch in Cyp33 to repress MLL1-mediated transcription. Sci Adv 9:eadf5330 10.1126/sciadv.adf5330 PMC10115415 37075125

[52] Manders EMM, Verbeek FJ, Aten JA (1993) Measurement of co-localization of objects in dual-colour confocal images. J Microsc 169:375–382 10.1111/j.1365-2818.1993.tb03313.x 33930978

[53] Costes SV et al (2004) Automatic and quantitative measurement of protein-protein colocalization in live cells. Biophys J 86:3993–4003 10.1529/biophysj.103.038422 PMC1304300 15189895

[54] Mocciaro E et al (2023) WDR5 is required for *DUX4* expression and its pathological effects in FSHD muscular dystrophy. Nucleic Acids Res 51:5144–5161 10.1093/nar/gkad230 PMC10250208 37021550

[55] Vangone A, Bonvin AM (2015) Contacts-based prediction of binding affinity in protein–protein complexes. Elife 4:e07454 10.7554/eLife.07454 PMC4523921 26193119

[56] Abraham MJ et al (2015) GROMACS: high performance molecular simulations through multi-level parallelism from laptops to supercomputers. SoftwareX 1–2:19–25

[57] Jo S, Kim T, Iyer VG, Im W (2008) CHARMM-GUI: a web-based graphical user interface for CHARMM. J Comput Chem 29:1859–1865 10.1002/jcc.20945 18351591

[58] Huang J et al (2017) CHARMM36m: an improved force field for folded and intrinsically disordered proteins. Nat Methods 14:71–73 10.1038/nmeth.4067 PMC5199616 27819658

