# Supplementary material for: The isolated HEPN domain of SACSIN exhibits RNA-binding activity
Source: Cell Mol Life Sci. 2026 Aug 27;83(1):378. doi: 10.1007/s00018-026-06369-w (PMC13669270; doi:10.1007/s00018-026-06369-w)
Supplement: Supplementary file 1 [file 18_2026_6369_MOESM1_ESM.pdf]

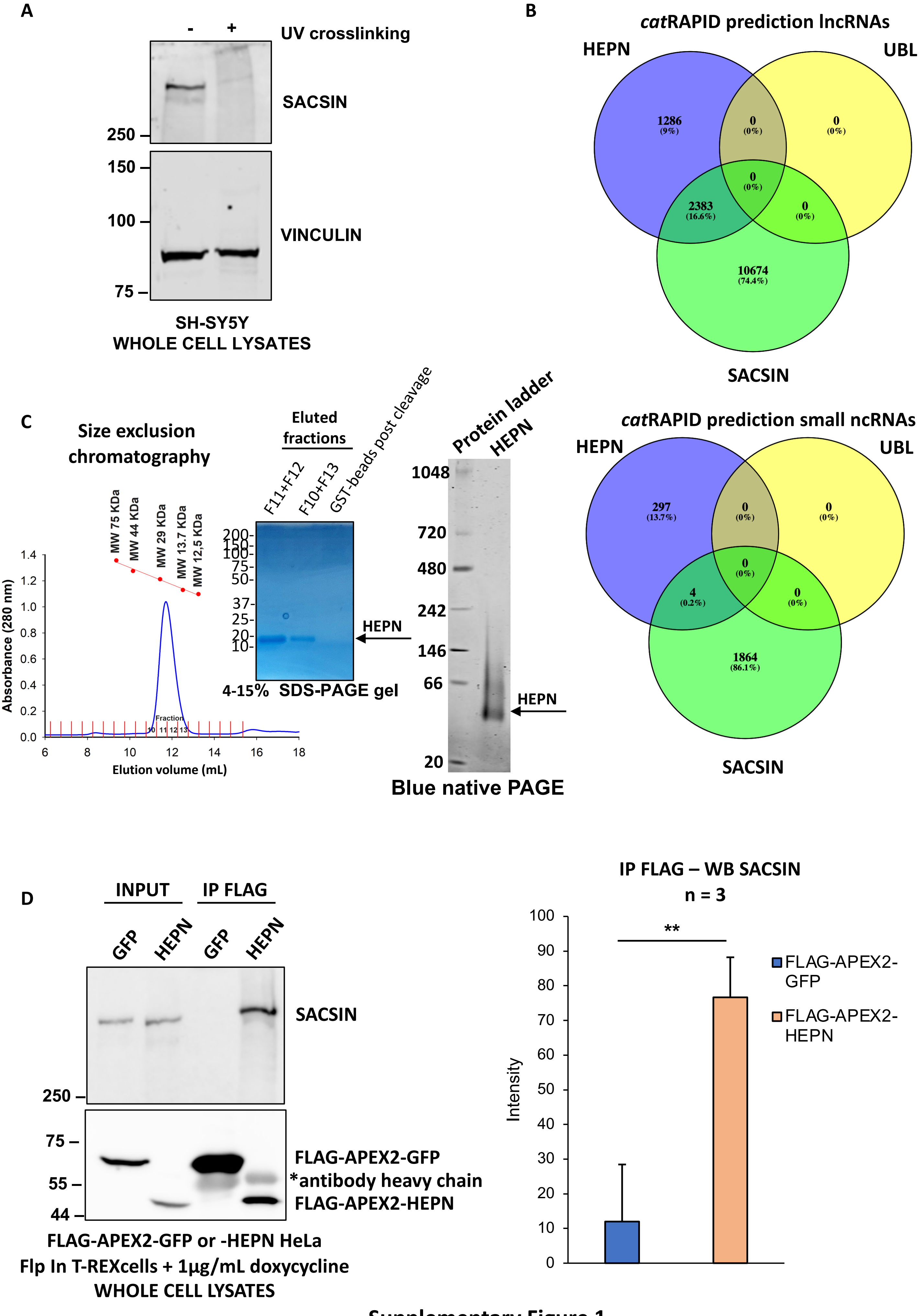

Supplementary Figure 1

A

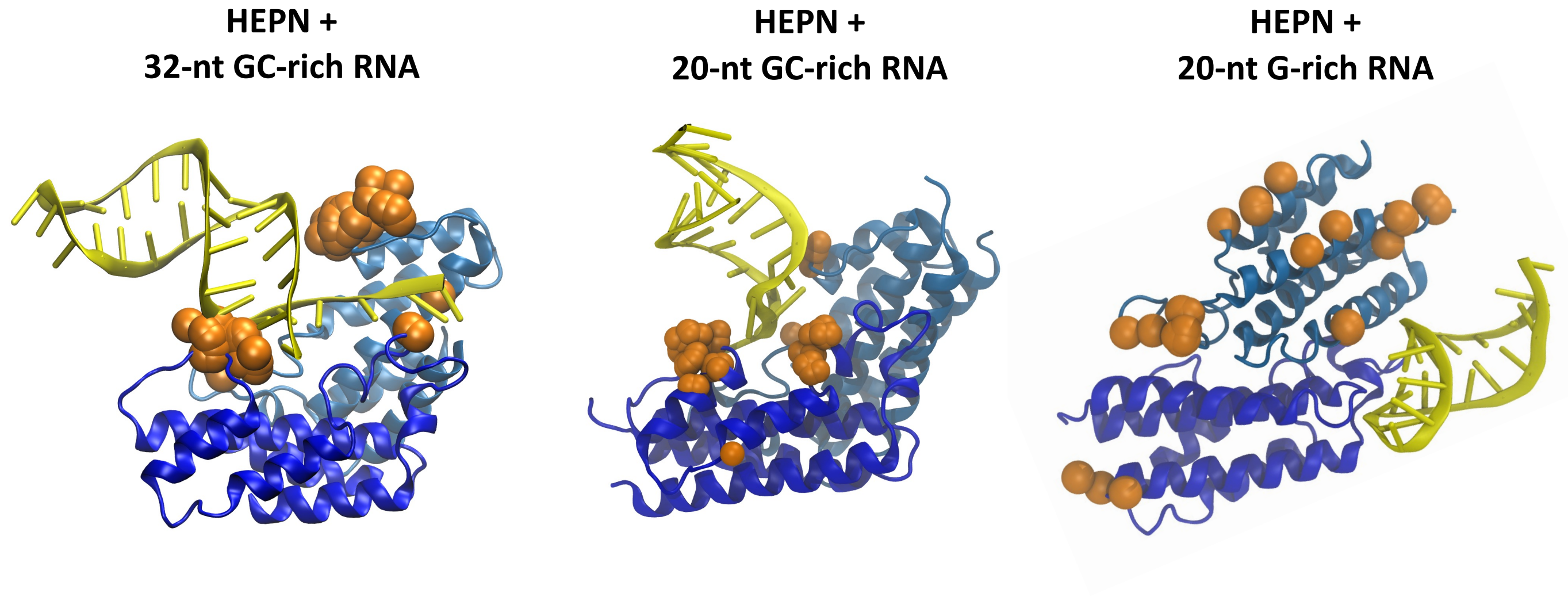

B

RNA-fold web server predictions

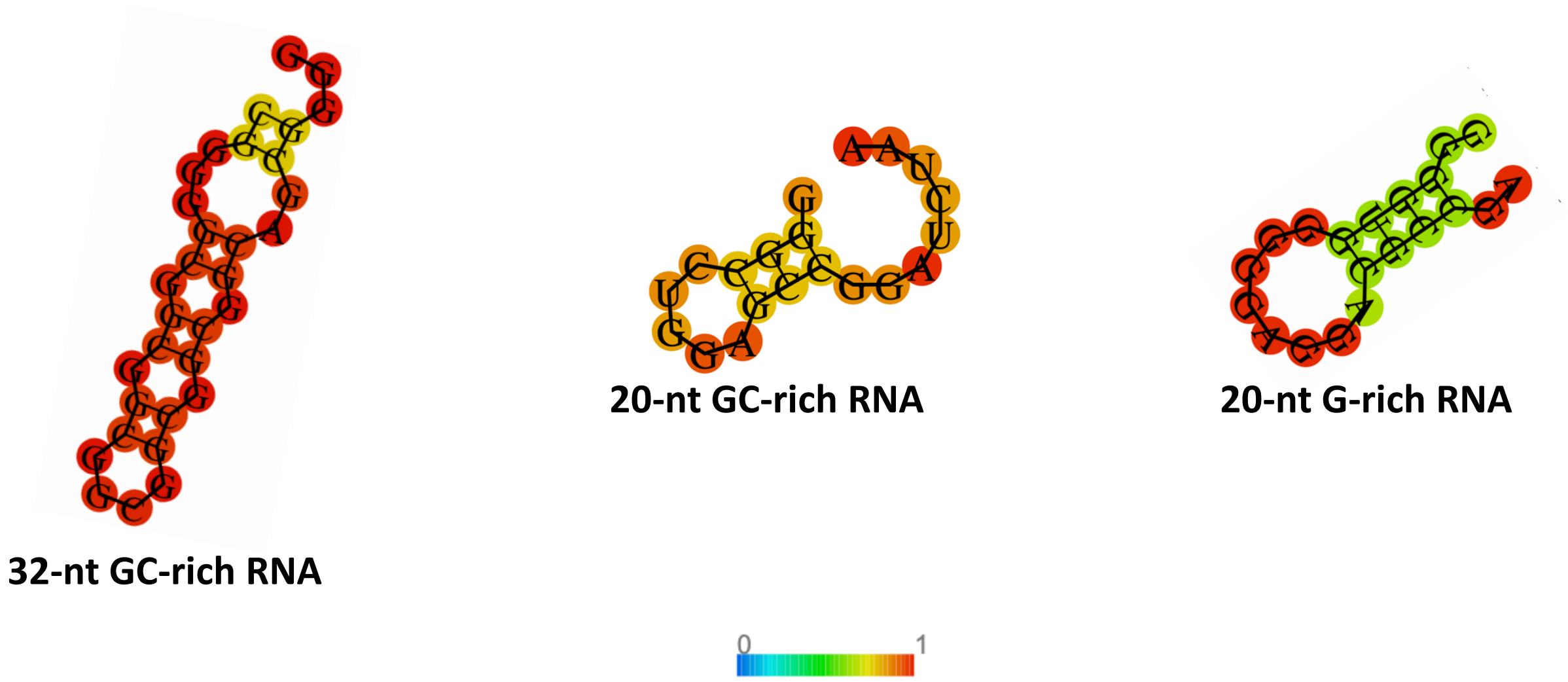

Supplementary Figure 2

A

Conc.(μM)	Response
10	2.16E-02
5	4.10E-02
2.5	3.66E-02
1.25	-1.69E-03
0.625	-2.45E-02
0.3125	-2.94E-02
0.1563	-2.97E-02
0.0781	-3.09E-02

B

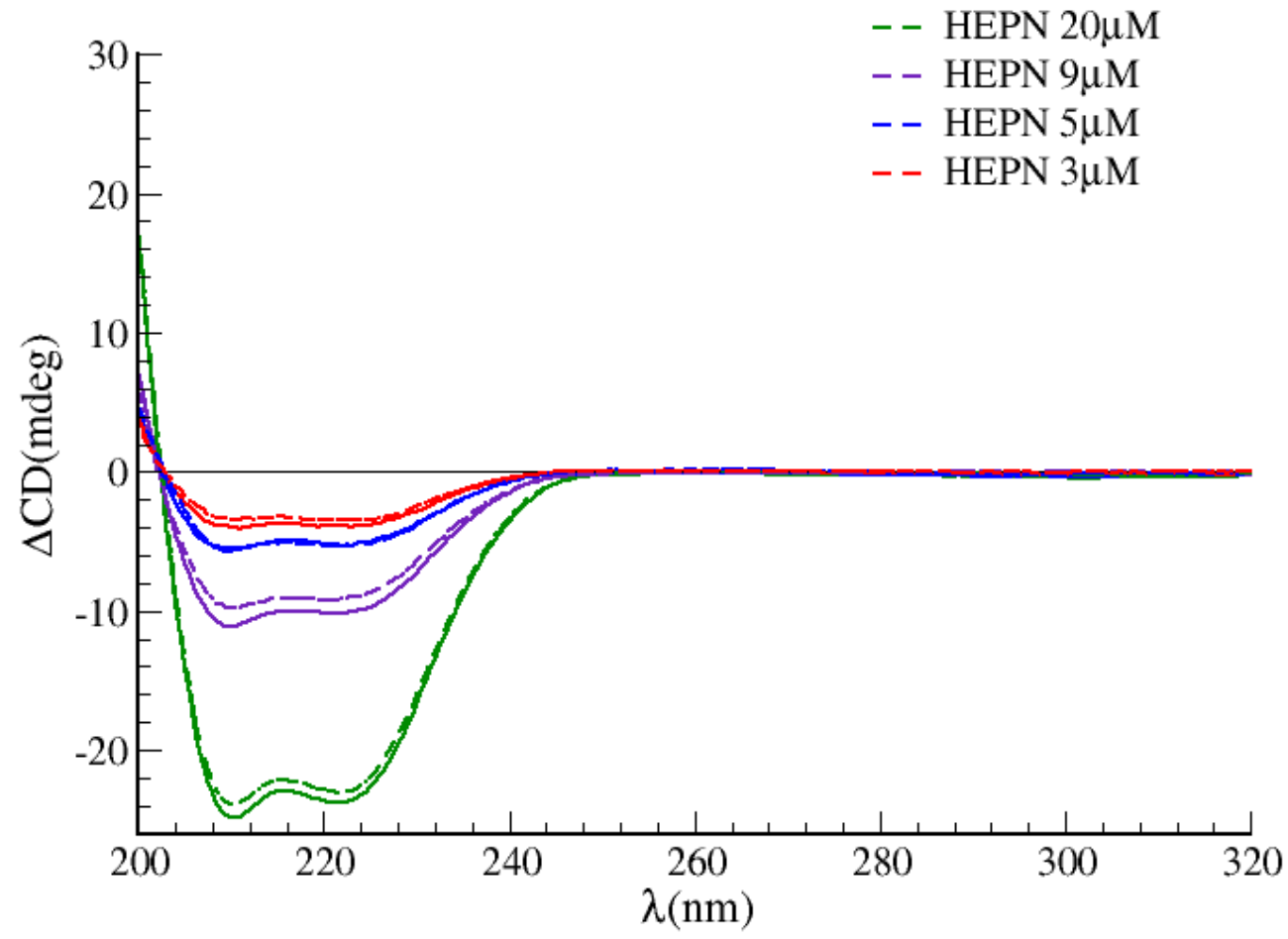

C

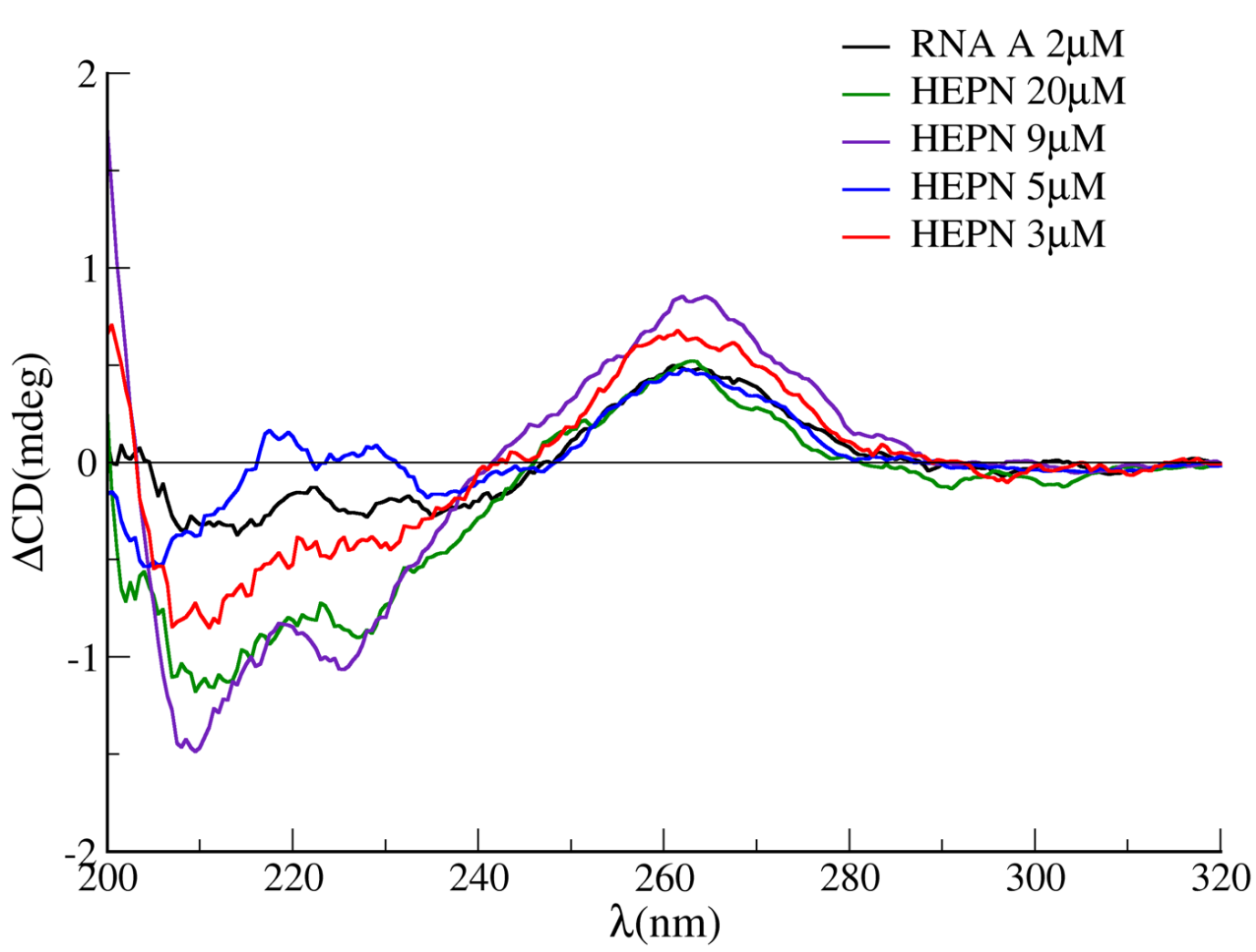

D

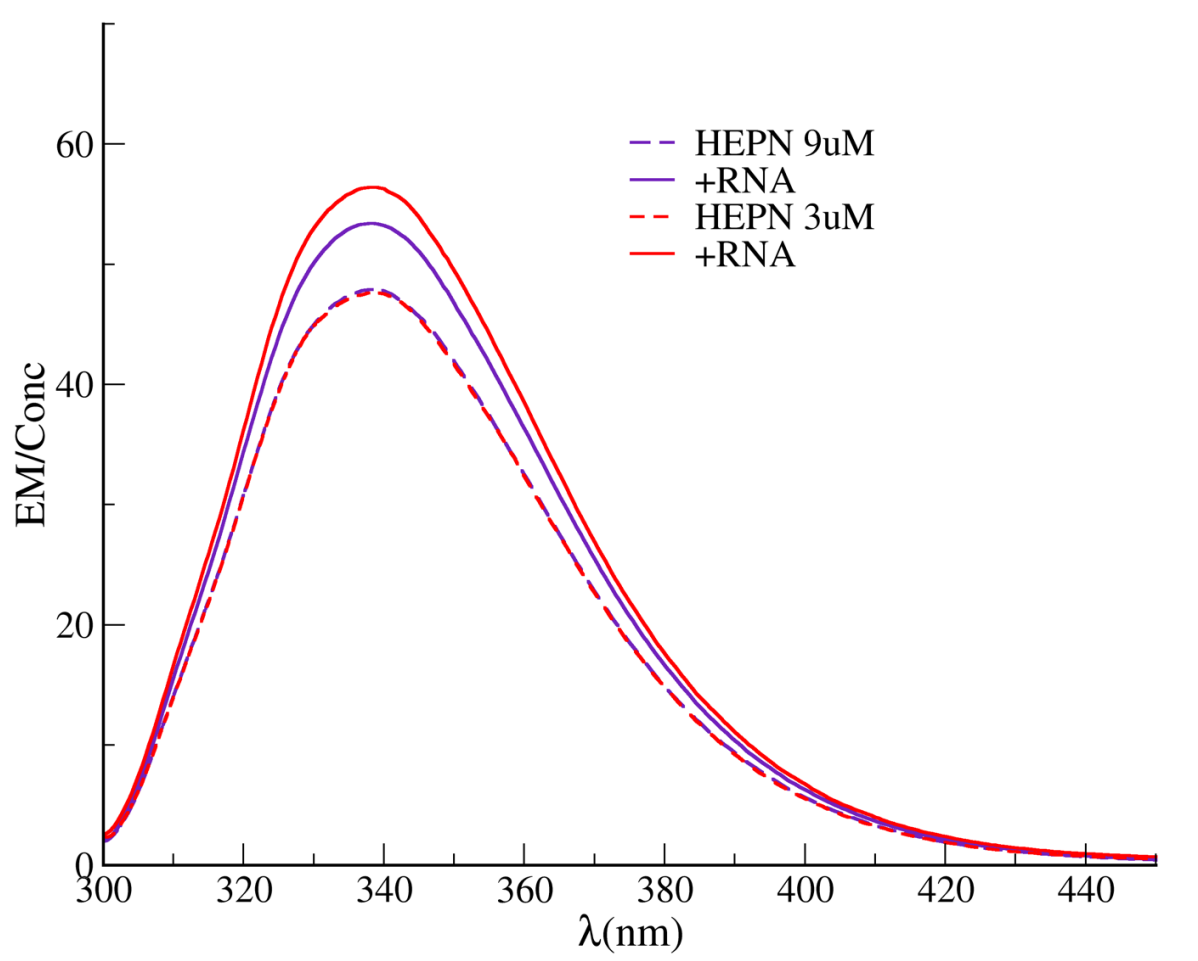

Supplementary Figure 3

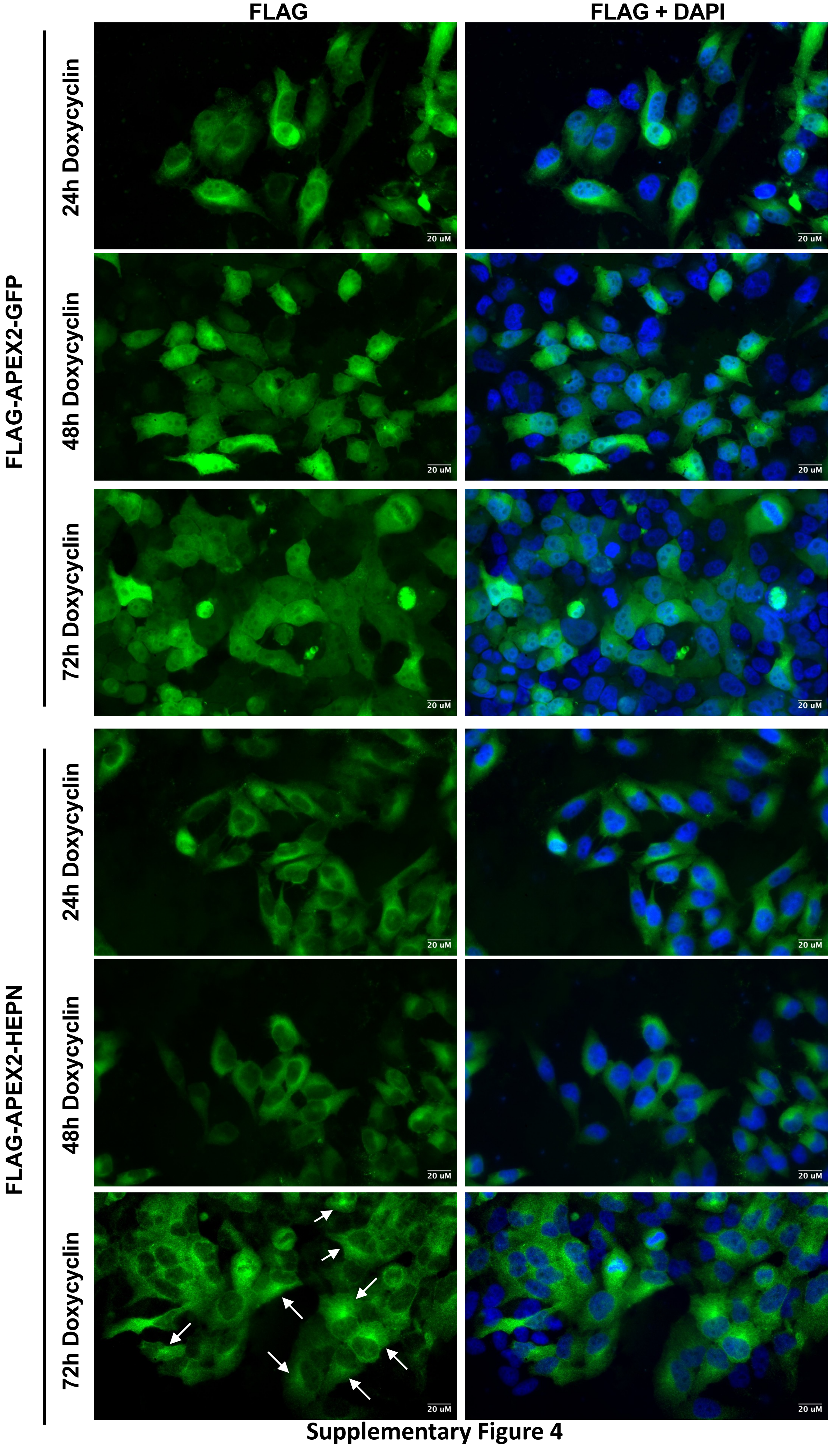

Supplementary Figure 4

A

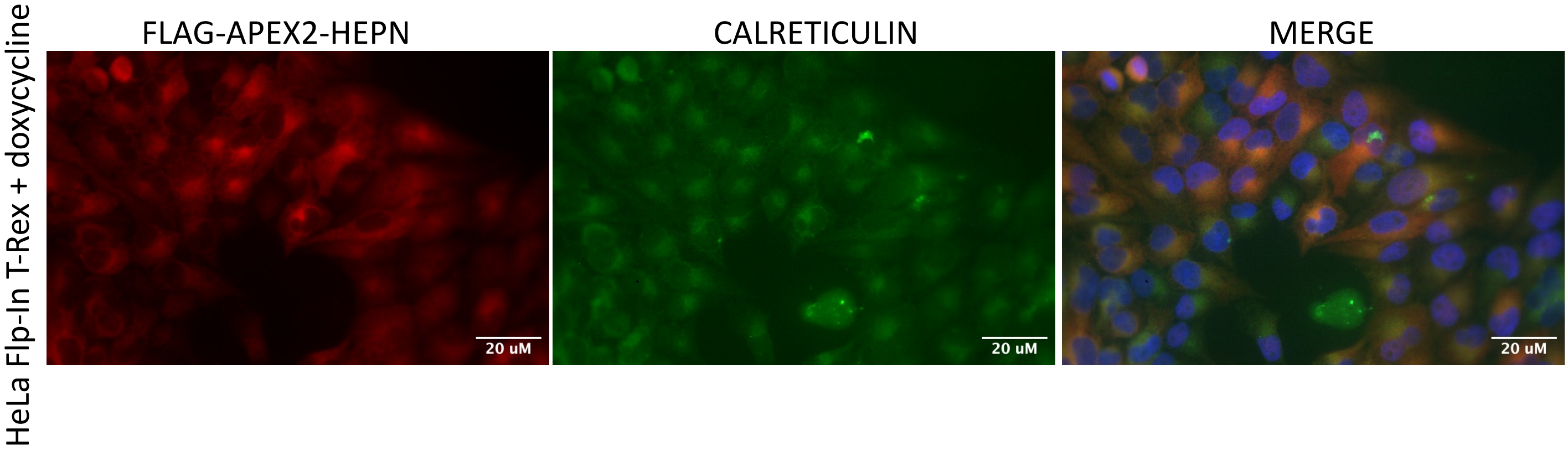

B

FLAG + GRP94	ROI
tM1	0.5791
tM2	0.6536
Costes observed r (block=3, n=100)	r=0.5081
Randomized r mean ± SD	0.0009 ± 0.0070
Costes significance	100.0 %
Monte Carlo p	0.0099

FLAG + CALRETICULIN	ROI
tM1	0.8309
tM2	0.8754
Costes observed r (block=3, n=100)	r=0.9016
Randomized r mean ± SD	0.0018 ± 0.0185
Costes significance	100.0 %
Monte Carlo p	0.0099

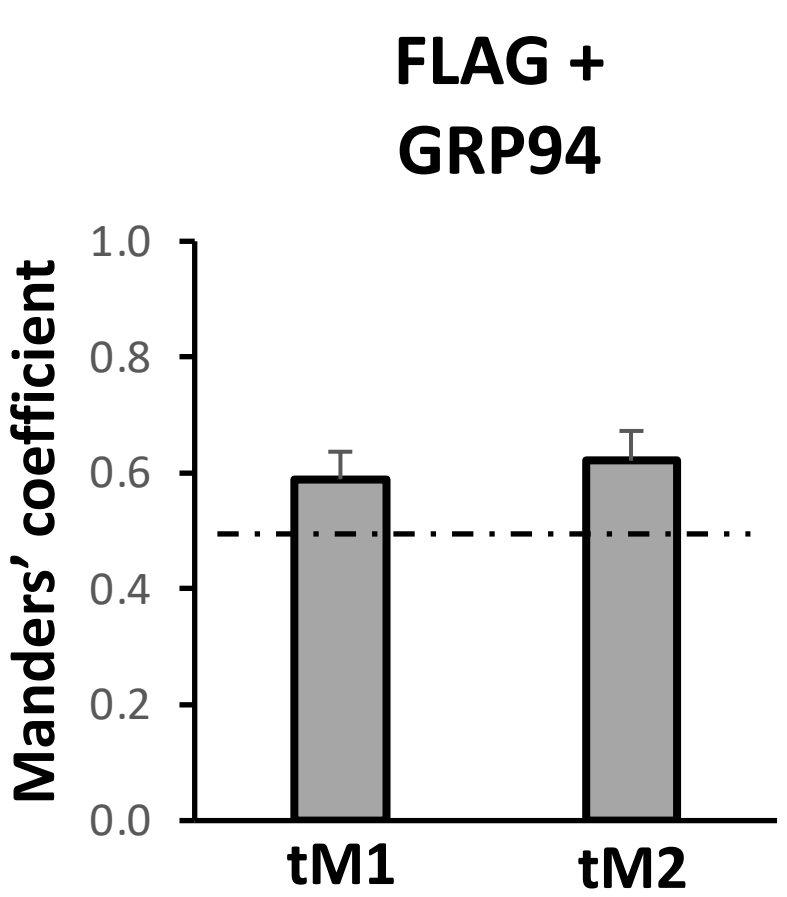

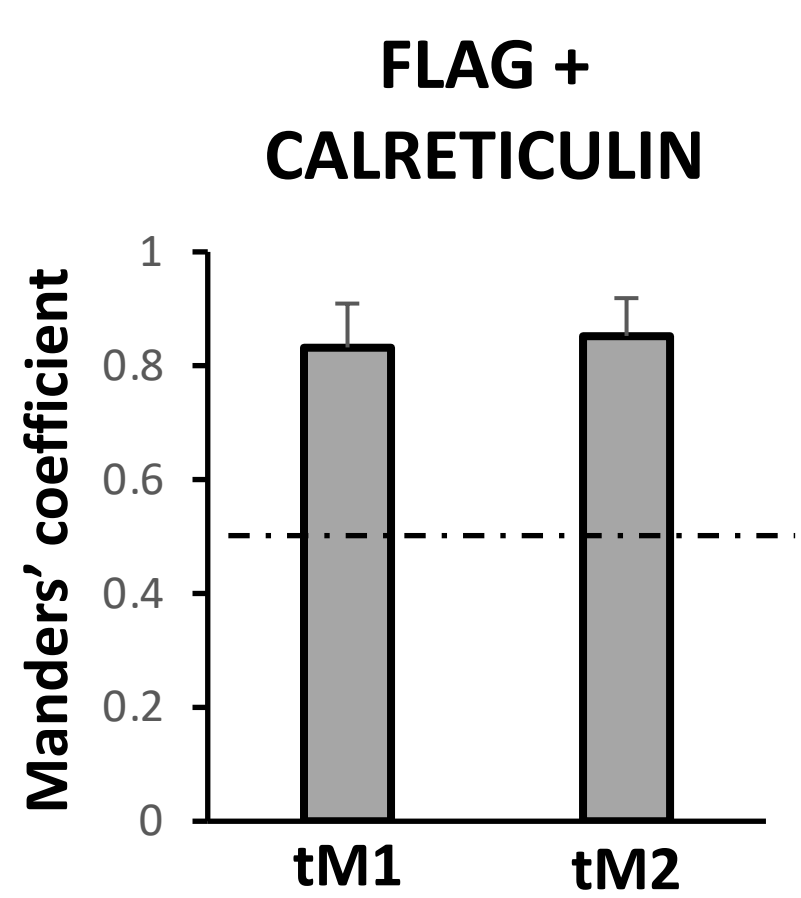

Supplementary Figure 5

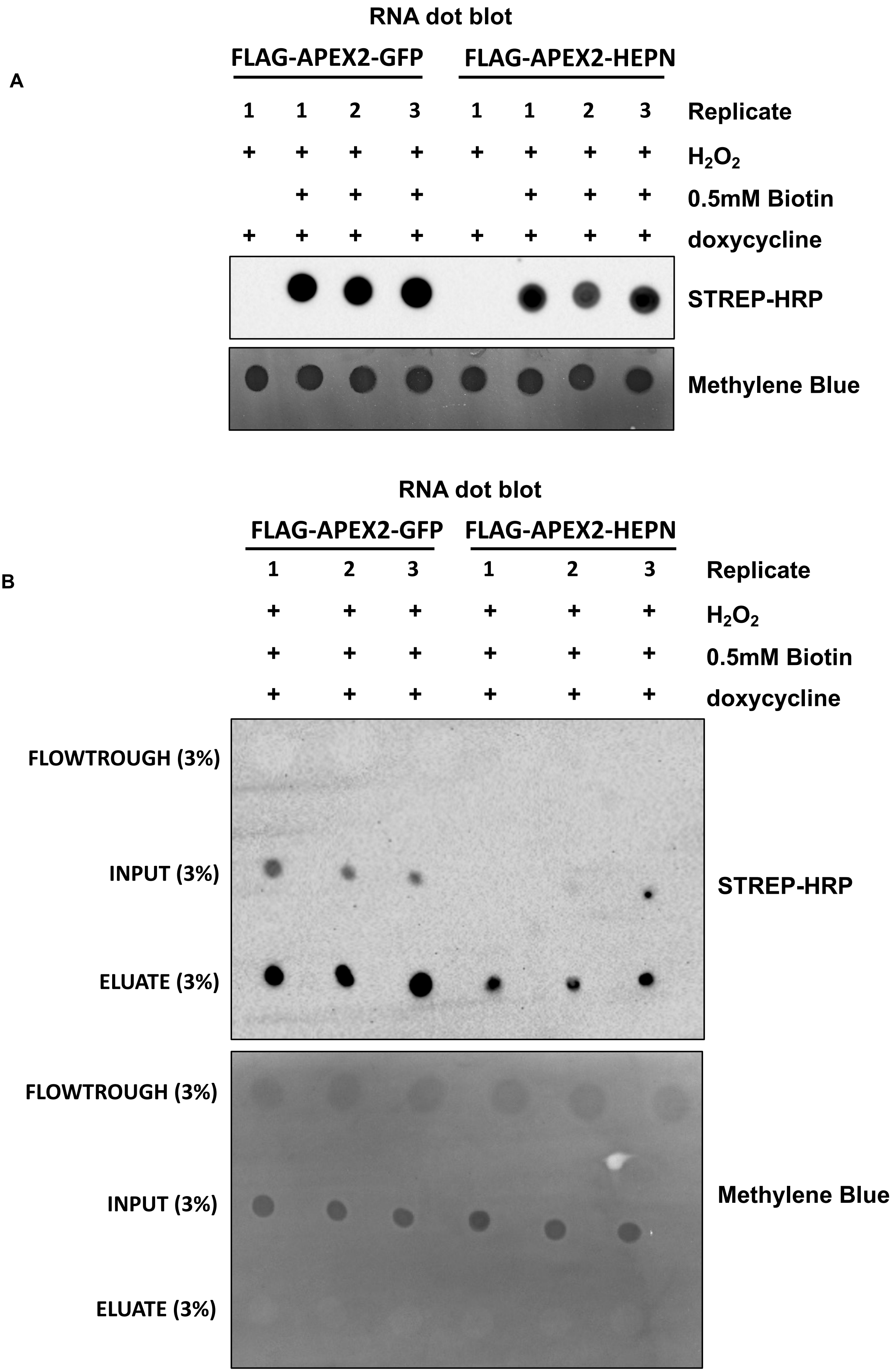

Supplementary Figure 6

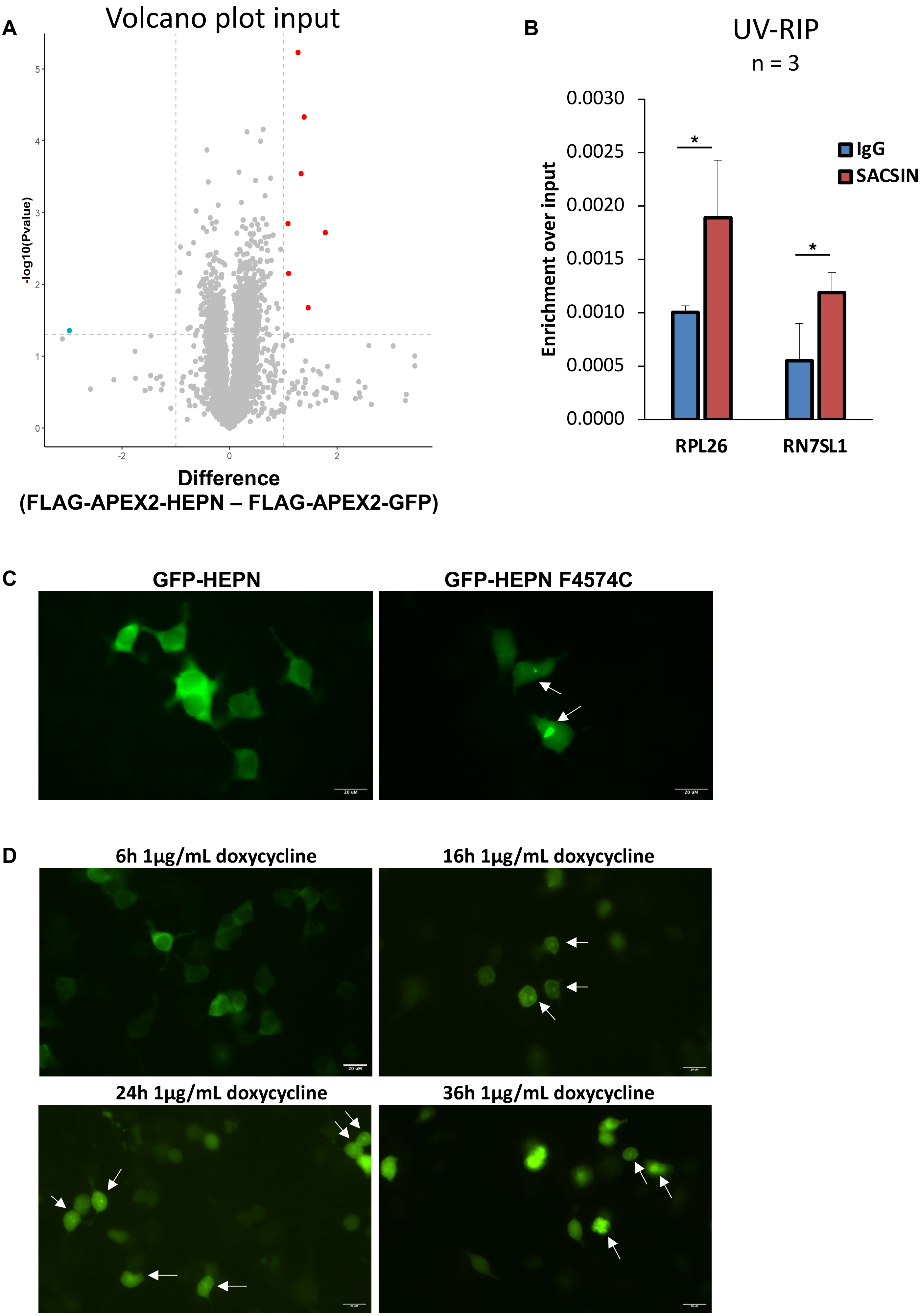

Supplementary Figure 7

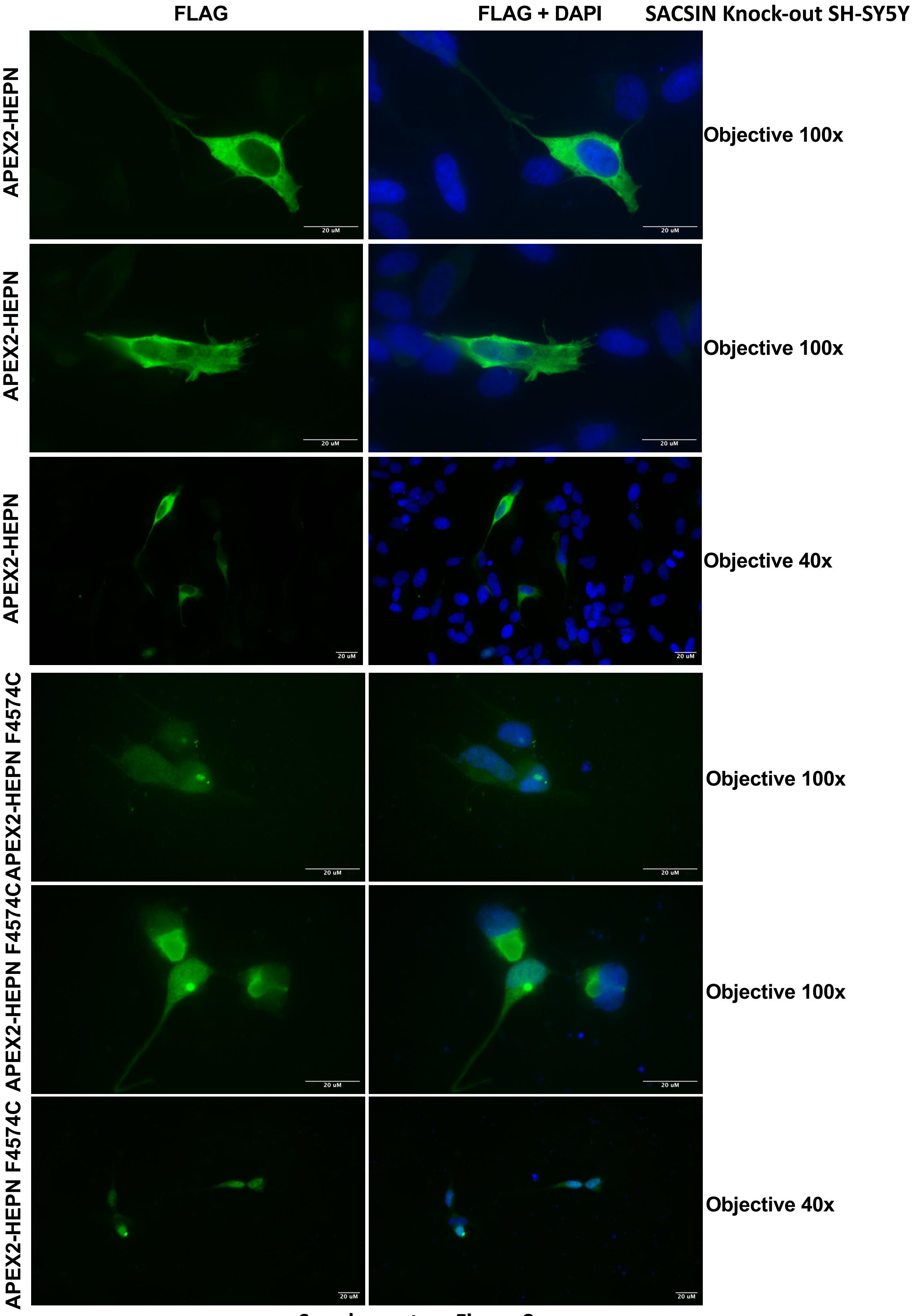

Supplementary Figure 8

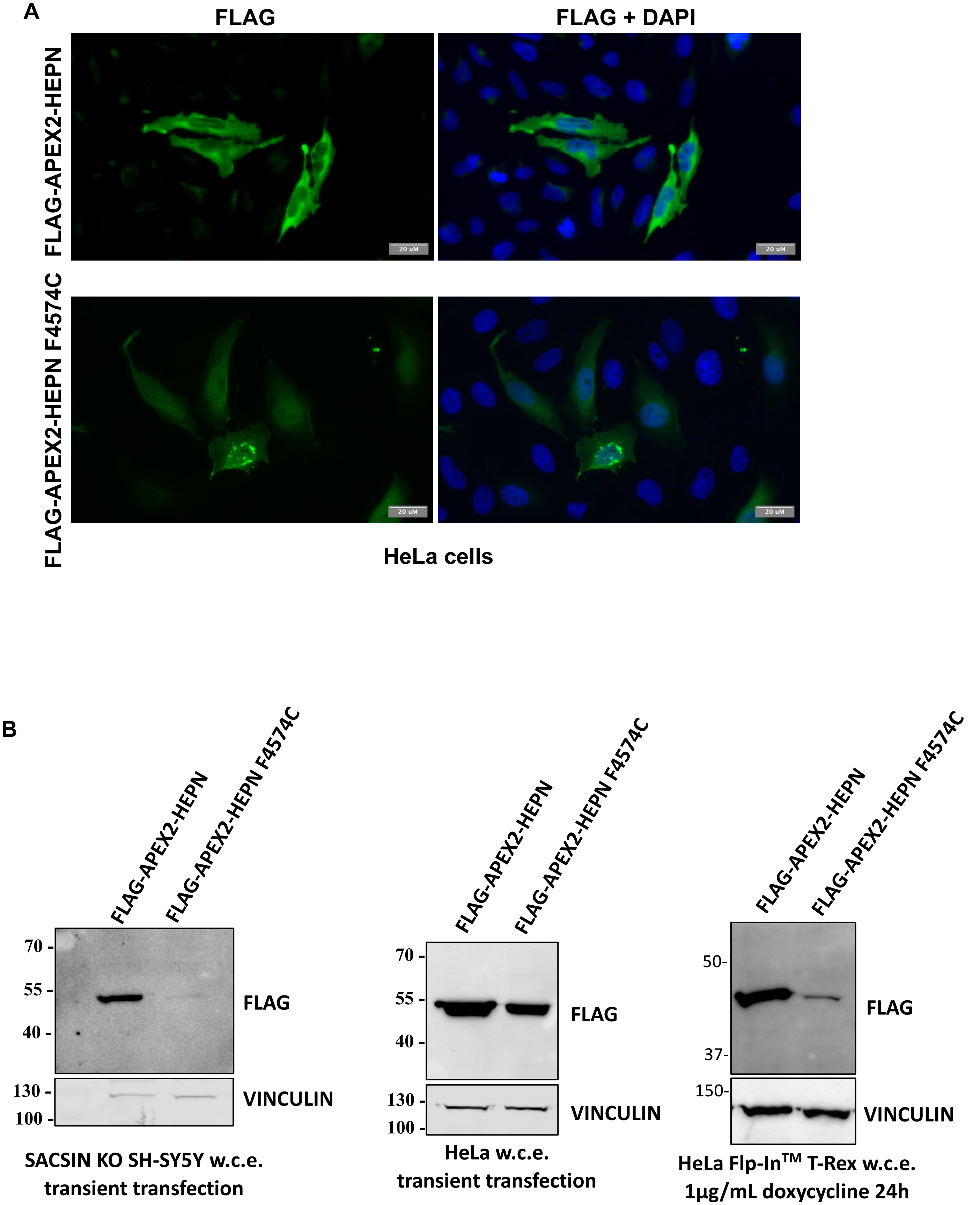

Supplementary Figure 9
