# Supplementary material for: The isolated HEPN domain of SACSIN exhibits RNA-binding activity
Source: Cell Mol Life Sci. 2026 Aug 27;83(1):378. doi: 10.1007/s00018-026-06369-w (PMC13669270; doi:10.1007/s00018-026-06369-w)
Supplement: Supplementary file 2 [file 18_2026_6369_MOESM2_ESM.docx]

RNA transcripts with a Z-score > 3.5 with both HEPN and SACSIN:

ENST00000327064 CARM1	45-176

>NM_199141.2:45-176 Homo sapiens coactivator associated arginine methyltransferase 1 (CARM1), transcript variant 1, mRNA

CGGCGGCGGCGGCGGCGGCGGCGGCGGCGGCGGCGGCGGCGGCAGCGGCGGCGGCCTGGGCCCGGGCGCA

GCGGCGGCGGCGGCGGGGCCTGGAGCCGGATCTAAGATGGCAGCGGCGGCGGCGGCGGTGGG

ENST00000359562 HAND2	800-911

>NM_021973.3:800-911 Homo sapiens heart and neural crest derivatives expressed 2 (HAND2), mRNA

GGCTGCCGGGCGGGCCCGGGCGCCCGGGGGCTGGGGGGTGGGGGGTGGGGGAGGACGCCGAGCGCTGAGG

CAGGGGCCCGGGCCGAGGGCGCGGCGGGGCTGCGCGCACGCT

ENST00000282111 TCF7L1	296-415

>NM_031283.3:296-415 Homo sapiens transcription factor 7 like 1 (TCF7L1), mRNA

GCCCCAGCTCGGCGGCGGGGGCGGCGGCGGCGGCGGCGGCAGCGGGGGAGGCGGCGGCTCCAGCGCCGGG

GCGGCCGGCGGAGGGGACGACCTCGGGGCGAACGACGAGCTGATCCCCTT

ENST00000373012 POU3F1	532-651

>NM_002699.4:532-651 Homo sapiens POU class 3 homeobox 1 (POU3F1), mRNA

AGGCGGCCTACCCGGGGGGCGGCGGCGGCGGCCTGGCCGGGATGCTGGCGGCGGGCGGTGGCGGCGCGGG

GCCGGGCCTGCACCACGCGCTGCACGAGGATGGCCACGAGGCGCAGCTGG

ENST00000222374 CADM4	01-88

>NM_145296.2:1-88 Homo sapiens cell adhesion molecule 4 (CADM4), mRNA

GGCGCGGGCGGTGCAGGTGCCGGGCCGGGAGCGAGCGGCGGCGGCGGCGGCGGCGGCACCATGGGCCGGG

CCCGGCGCTTCCAGTGGC

ENST00000326873 STK11	303-434

>NM_000455.5:303-434 Homo sapiens serine/threonine kinase 11 (STK11), transcript variant 1, mRNA

GGACGCTGAGGGCACTCGGGGCGGGGCGCGCGCTCGGGCAGACGTTTGCGGGGAGGGGGGCGCCTGCCGG

GCCCCGGCGACCACCTTGGGGGTCGCGGGCCGGCTCGGGGGGCGCCCAGTGCGGGCCCTCGC

ENST00000302101 NHLH1	517-620

>NM_005598.4:517-620 Homo sapiens nescient helix-loop-helix 1 (NHLH1), mRNA

CTGTGGGGGCGGGGCGGGCCCTGATGGTGCCGGGCCTGGGGGTCCGGGAGGGGGCCAGGCCCGAGGCCCA

GAGCCGGGAGAGCCTGGCCGGAAAGACCTGCAGC

ENST00000308219 BRSK2	201-364

>NM_001256627.1:201-364 Homo sapiens BR serine/threonine kinase 2 (BRSK2), transcript variant 1, mRNA

GCCGCGTCGGAGTGGACGCGGGGGGCGGCGGCGCGGGCGGACGCGGGCGGCGCGAAGCAGCGGGGCCCGC

GGGGGCGCCCCGGCCGGGTCGGCGCGGACGGCACTCGGCGGACGCGGGCGGACGCTGGGCGGCCCCTCCC

TGCCCGCGCGCCCGGGCGCCCCTG

RNA transcripts with a Z-score < 0 with both HEPN and SACSIN:

> ENST00000335185	CCDC73	2889-3042

aacaaaaagatcatttcaatggctctttgtaaaaatattggtgtggatgatgttggaaaggatattggaccagatactacca gcattaacagagttgctgacactttgaataactggagtatccatccagatcccaagggagaacccagtgaag

> ENST00000304402	GPR22	1-120

gtcattctgcaaaagctgcccgaacaggct ttcctctgaa aagcagtgcc gttcctttttagaaagactg aatctaaatg ttgtctctgg agacaagaagccttcagtatgttaaattac

> ENST00000226730	IL2	51-102

cattcagtcagtctttgggggtttaaagaaattccaaagagtcatcagaaga

Clustal Omega alignment output
